# Viability of Wildflower Seeds After Mesophilic Anaerobic Digestion in Lab-Scale Biogas Reactors

**DOI:** 10.3389/fpls.2022.942346

**Published:** 2022-07-14

**Authors:** Juliane Hahn, Paula R. Westerman, Friederike de Mol, Monika Heiermann, Bärbel Gerowitt

**Affiliations:** ^1^Crop Health, Faculty of Agricultural and Environmental Sciences, University of Rostock, Rostock, Germany; ^2^Department Technology Assessment and Substance Cycles, Leibniz Institute for Agricultural Engineering and Bioeconomy (ATB), Potsdam, Germany

**Keywords:** dose response models, exposure time, flowering wild plant mixtures, hardseededness, physical dormancy, seed survival, seed viability, temperature

## Abstract

The use of wildflower species as biogas feedstock carries the risk that their seeds survive anaerobic digestion (AD) and cause weed problems if spread with the digestate. Risk factors for seed survival in AD include low temperature, short exposure and hardseededness (HS). However, it is not possible to predict how AD will affect seed viability of previously unstudied species. In laboratory-scale reactors, we exposed seeds of eight species from a mixture of flowering wild plants intended as biogas feedstock and three reference species to AD at two mesophilic temperatures. Half of the species were HS, the other was non-HS (NHS). Viability was determined using a combination of tetrazolium and germination tests. Viability and germinability were modeled as functions of exposure time using a dose-response approach. Responses to AD varied considerably among species, and none of the considered influencing factors (time, temperature, HS) had a consistent effect. Seed lots of a species differed in inactivation times and seed-killing efficacy. The HS species *Melilotus officinalis*, *Melilotus albus*, and *Malva sylvestris* were particularly AD-resistant. They were the only ones that exhibited biphasic viability curves and tended to survive and germinate more at 42°C than at 35°C. Viability of the remaining species declined in a sigmoidal curve. Most NHS species were inactivated within a few days (*Cichorium intybus*, *Daucus carota*, *Echium vulgare*, and *Verbascum thapsus*), while HS species survived longer (*Malva alcea*). AD stimulated germination in the HS species *A. theophrasti* and its AD-resistance overlapped with that of the most resistant NHS species, *C. album* and tomato. In all seed lots, germinability was lost faster than viability, implying that mainly dormant seeds survived. After the maximum exposure time of 36 days, seeds of HS species and *Chenopodium album* were still viable. We concluded that viability responses to mesophilic AD were determined by the interplay of AD-conditions and species- and seed-lot-specific traits, of which HS was an important but only one factor. For the use of wildflowers as biogas feedstock, we recommended long retention times and special care with regard to HS species.

## Introduction

Anaerobic digestion (AD) of renewable feedstocks in biogas plants is considered one of the most environmentally friendly and energy efficient bioenergy sources. A particular advantage is that the semi-solid leftover of biogas production, the digestate, which is produced in addition to the energy carrier methane, can be used as plant fertilizer, thus tightening nutrient cycles (Weiland, 2010; Guo et al., 2015; Salnikova et al., 2019). AD feedstocks have included energy crops such as maize (*Zea mays* L.), triticale (× *Triticosecale*) or beets (*Beta vulgaris* L.) for decades (e.g., Venendaal et al., 1997; Herrmann et al., 2016; Hofmann et al., 2017). The sustainability of using energy and agrofuel crops, however, has become increasingly controversial (e.g., Altieri, 2009; Eggers et al., 2009; Meyer-Aurich et al., 2012; Gasparatos et al., 2013). In response, there are now calls for biomass production systems to be multifunctional and adapted to local conditions (von Cossel et al., 2019b; Englund et al., 2020b). For this reason, among others, the portfolio of energy crops continues to expand (e.g., Papamatthaiakis et al., 2021). The focus is on perennial species that offer a variety of ecological benefits at low input (Don et al., 2012; Müller-Stöver et al., 2016; Emmerling et al., 2017; Hofmann et al., 2017; Jones, 2017; Englund et al., 2020a). Probably the most diverse option at present is the cultivation of perennial species mixtures (e.g., von Cossel and Lewandowski, 2016; Carlsson et al., 2017; Weißhuhn et al., 2017; Yang et al., 2018).

Since 2008, mixtures of flowering wild plant species were introduced in Germany to supplement silage maize as a biogas feedstock (Vollrath, 2012; Vollrath et al., 2016; von Cossel and Lewandowski, 2016). These flowering mixtures are of interest mainly because they significantly improve ecosystem services such as habitat functioning, soil protection and landscape aesthetics (von Cossel, 2020; Janusch et al., 2021), while their methane yield is rather low (von Cossel et al., 2019a,^2021^; Lask et al., 2002). However, a sustainability issue rarely considered is that wildflower mixtures and other energy crops can spread undesirably into new habitats. When used as a biogas feedstock, propagules such as seeds that survive the biochemical processes during AD enter the digestate. There is a risk that these establish as weeds on fields fertilized with this digestate. Of course, seed persistence in soil is only one of many criteria for weediness (Baker, 1974), however, according to Harper (1977) it is important for the success of plants in farmed fields. The weed control measures required upon seed survival would compromise sustainability and cause undesirable, additional costs and labor. Non-native or quarantine species not yet widespread are particularly problematic in this context (Raghu et al., 2006; Simberloff, 2008; Westerman and Gerowitt, 2013). In this regard, the biogas wildflower mixtures should be evaluated with care, as they contain various poorly cultivated (wild) species. However, whether seeds of species from biogas wildflower mixtures survive AD has not yet been the subject of investigations. In general, studies on seed susceptibility to AD are scarce and systematic studies on the ability of seeds from different taxonomic and functional groups to survive AD are lacking (Westerman and Gerowitt, 2013). Thus, reliable predictions of seed viability of previously unstudied plant species in AD are not possible.

Most available studies on seed survival in AD dealt with weeds (e.g., Jeyanayagam and Collins, 1984; Šarapatka et al., 1993; Schrade et al., 2003; Eckford et al., 2012; Westerman et al., 2012a; Johansen et al., 2013; Zhou et al., 2020). Of the plants whose biomass is (intended to be) used as biogas feedstock, only 14 species have been studied to date (Heiermann et al., 2010; Strauß et al., 2012; van Meerbeek et al., 2015; Baute et al., 2016; Sölter et al., 2016; Starfinger and Sölter, 2016; Hassani et al., 2021). Based on weeds studied through 2012, Westerman and Gerowitt (2013) identified plant groups whose seeds might have a higher probability of surviving AD than usual. They comprised species that are either hardseeded (HS), i.e., form physically dormant seeds with one or more impermeable layers in the seed or fruit coat (Baskin et al., 2000), and species adapted to dispersal by endozoochory, e.g., by thick seed coats. HS is common in the Fabaceae and occurs in members of the Malvaceae (Baskin et al., 2000), both of which are families of interest for biogas flowering mixtures (Vollrath, 2012). HS as a risk factor for high AD-resistance potential and consequently for seed dispersal with the digestate has been explicitly mentioned by Leonhardt et al. (2010), Westerman et al. (2012a,b), and Hassani et al. (2021). However, not all species resistant to AD are HS, so it is suspected that other seed traits may aid seed survival in AD as well (Westerman and Gerowitt, 2013).

In addition to characteristics of the seeds themselves, temperature and exposure time were found to be the most important factors driving seed inactivation in AD. In general, seed viability decreases exponentially with time, with the seeds remaining unaffected by AD during an initial lag-phase (Westerman and Gerowitt, 2013). In addition, higher temperatures result in a greater decrease in seed viability (reviewed by Westerman and Gerowitt (2013) and confirmed by Johansen et al. (2013); Oechsner et al. (2018), and Zhou et al. (2020). In particular, ADs under thermophilic conditions (approx. 45–55°C) appear to be significantly more effective in killing seeds than mesophilic ones (approx. 30–45°C) (Šarapatka et al., 1993; Lorenz et al., 2001; Schrade et al., 2003; Westerik and Kleizen, 2006; Leonhardt et al., 2010; Johansen et al., 2013; Zhou et al., 2020). This implies that ADs in the mesophilic temperature range pose a higher risk of unintended seed spread – as pointed out by Westerman and Gerowitt (2013); Alsanius et al. (2021), and Hassani et al. (2021). With regard to the use of wildflower species as biogas feedstock, this could be problematic, as they are to be grown mainly in Germany, where 84% of biogas plants are mesophilic (vTI, 2009).

Finally, existing studies differ in which seeds they consider viable. Many studies on the effects of AD on seed viability have based their conclusions solely on germination tests (Engeli et al., 1993; Lorenz et al., 2001; Ryckeboer et al., 2002; Schrade et al., 2003; Marcinisyn et al., 2004; Westerik and Kleizen, 2006; Strauß et al., 2012; Johansen et al., 2013; Milotić and Hoffmann, 2016; Oechsner et al., 2018; Zhou et al., 2020). In doing so, they did not consider that dormant seeds may have survived and could germinate once dormancy is broken. However, HS species and wild plant species in general can exhibit different classes, levels, and types of dormancy (Baskin and Baskin, 1998, 2004). Therefore, to determine the actual risk of spreading viable seed with the digestate, the (total) viability must be determined as the sum of germinable and dormant seeds. This procedure has only been used in some studies on seed survival in AD (Jeyanayagam and Collins, 1984; Eckford et al., 2012; Westerman et al., 2012a,b; Baute et al., 2016).

The objective of this study was to evaluate the effects of mesophilic AD on seed viability of wildflower species intended as biogas feedstocks. In addition, three species that have already been investigated in similar studies were included as references. The focus was on the impact of AD-process control parameters on seed viability of hardseeded (HS) or non-hardseeded (NHS) species. Seed viability was explored as a function of exposure time in AD at two mesophilic temperatures. We hypothesized that in AD, (1) seed viability of species with HS would be reduced less than that of NHS species, (2) seed viability would decrease more at higher incubation temperatures, and (3) seed viability would decrease with increasing exposure time. In addition, for both HS and NHS species, we examined whether seeds that survived AD were germinable or dormant. Finally, we discussed the implications of the results with respect to the use of wildflower species as biogas feedstocks.

## Materials and Methods

### Plant Species

#### Species Selection

Seed vitality after mesophilic anaerobic digestion (AD) was studied in eleven different species. Five of the species were hardseeded (HS) the others not (NHS). The majority of species were selected from a wildflower mixture that has been specifically designed for biogas production (‘‘BG70’’ by Saaten Zeller GmbH & Co. KG, Eichenbühl-Guggenberg, Germany).^1^ From this mixture, *Malva alcea* L. (rose mallow, Malvaceae), *Malva sylvestris* L. (common mallow, Malvaceae), *Melilotus albus* MEDIK. (white sweet clover, Fabaceae) and *Melilotus officinalis* (L.) PALL. (yellow sweet clover, Fabaceae) were selected to represent HS species. NHS representatives were *Cichorium intybus* L. (Blue dandelion, Asteraceae), *Daucus carota* L. (wild carrot, Apiaceae), *Echium vulgare* L. (viper’s bugloss, Boraginaceae) and *Verbascum thapsus* L. (great mullein, Scrophulariaceae). In selecting NHS species, emphasis was placed on ensuring that they were from diverse families whose response to mesophilic AD has been poorly investigated.

In addition to the eight flowering species from the biogas mixture, this study included one HS and one NHS weed species that were found to be relatively resistant to mesophilic AD. *Abutilon theophrasti* MEDIK. (Malvaceae, velvetleaf) is a HS species whose seeds that survived AD with relatively high probability (Katovich et al., 2004; Westerman et al., 2012a,b). The NHS species *Chenopodium album* L. (Amaranthaceae, common lambsquarters) and tomato (*Lycopersicon esculentum* Mill., Solanaceae) were among the best surviving NHS species in several AD-treatments (Engeli et al., 1993; Šarapatka et al., 1993; Lorenz et al., 2001; Schrade et al., 2003; Katovich et al., 2004; Westerik and Kleizen, 2006; Leonhardt et al., 2010; Strauß et al., 2012; Westerman et al., 2012a,b; Johansen et al., 2013; Baute et al., 2016; Zhou et al., 2020).

#### Seed Lots, Seed Acquisition, and Storage

The following species were tested using only one seed lot: *D. carota*, *E. vulgare*, and *M. sylvestris* that were propagated in 2015 and obtained from ‘‘Herbiseed’’ (Twyford, United Kingdom, herbiseed.com). Seeds of *C. intybus*, *M. albus*, *M. officinalis* and *V. thapsus* were propagated in 2014 by ‘‘Appels Wilde Samen GmbH’’ (Darmstadt, Germany).^2^ Seeds of *C. album* were harvested in 2014 from *C. album* plants grown at the University of Rostock (Germany).

Two seed lots each were examined of *M. alcea*, *A. theophrasti* and tomato because one lot ran out during the course of the experiments, so a second lot was needed to obtain results for each species in all AD-treatments. Seeds of ‘‘*M. alcea -- 2 years*’’ and ‘‘*M. alcea -- 1 year’’* were ordered from ‘‘Appels Wilde Samen’’ (see above) and were propagated in 2014 and 2015, respectively. The seed lot ‘‘*A. theophrasti -- 7 years’’* was propagated and collected in 2008 in a sunflower field in Vilanova de Bellpuig, Lleida (Spain, collector PW). In 2015, the younger lot ‘‘*A. theophrasti -- 1 year’’* was propagated from seeds of ‘‘*A. theophrasti* -- *7 years’’* in a greenhouse at the University of Rostock (Germany). The seed lots of tomato came from two different varieties: ‘‘paprikaförmige’’ [‘tomato -- PAPRIKA’, propagated in 2014, ‘‘Culinaris -- Saatgut für Lebensmittel’’ (Göttingen, Germany)]^3^ and ‘‘St. Pierre’’ [‘tomato -- PIERRE,’ propagated in 2015, ‘‘Bingenheimer Saatgut AG’’ (Echzell, Germany)]^4^.

Until the beginning of and during the experiments in 2015, seeds were stored at room temperature in the dark. The seeds of “*A. theophrasti – 7 years*” harvested in 2008 had previously been stored at 7°C.

### Anaerobic Digestion of Seeds

#### Lab-Scale Reactors

**FIGURE 1:**
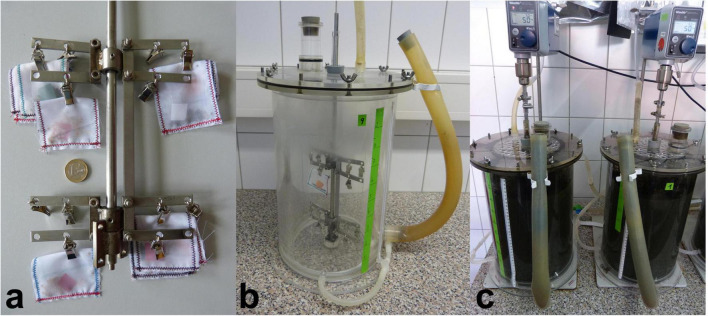
Experimental setup for anaerobic digestion of seeds in lab-scale reactors. **(a)** Central paddle stirrer with attached fine mesh polyester bags enclosing seeds and identification markers. **(b)** Lab-scale reactor consisting of a double-walled cylinder for temperature control via a water jacket, a gas-tight lid with tubes for feeding, gas collection, and attachment of the stirrer, and a separate outlet for the digestate at the bottom. **(c)** Running reactors connected to thermostat, stirrer drive, gas meter and gas analyzer.

From March 2015 until September 2016 seeds were exposed to mesophilic AD in eight lab-scale continuously stirred biogas reactors at the ATB in Potsdam (Germany) (Figure 1). The reactors were run according to German Standard Procedure VDI 4630 (VDI-Fachbereich Energietechnik, 2006). They had a working volume of 8 l each and were operated at a constant organic loading rate of 3 g_*VS*_ l^–1^ d^–1^ fed with maize silage and cattle slurry. Half of the reactors were run at 35°C, while the other half was run at 42°C, representing the lower and upper mesophilic temperature range of agricultural biogas plants (Weiland, 2010). In order to check the stability of the anaerobic digestion process the biogas produced was continuously measured with a milligascounter type TGC1/5 (RITTER, Bochum, Germany). Biogas volume measured was normalized to standard conditions: dry gas, t_0_ = 273 K, p_0_ = 1,013 hPa. The biogas composition was determined online using a gas analyzer SSM 6000 (PRONOVA, Berlin, Germany). To evaluate the stability of the biological process, samples of process liquid of each reactor were taken once a week and analyzed for total solids, volatile solids, ammonium nitrogen, total nitrogen, volatile fatty acids and pH (Terboven et al., 2017; Supplementary Data Sheet 1).

Continuous biogas production was monitored at both temperatures during seed exposure experiments. The average biogas production in the twelve reactors was 13.1 ± 2.61 l_*N*_ d^–1^ and the methane content was 57 ± 2% (data not shown). The actively proceeding biomethanation process was not disturbed by insertion and removal of the seeds (Supplementary Data Sheet 1).

#### Exposure of Seeds to Anaerobic Digestion

Seeds were placed in fine-mesh polyester bags attached to the reactors’ stirrer for exposure to AD and subsequent removal (Figure 1). Seeds were sampled after four different exposure times, and the duration of exposure differed between HS and NHS species. The seeds of NHS species were exposed to AD for 1, 3, 6, and 9 days at both 35 and 42°C. The species *C. album* and tomato were additionally exposed to 35°C for 18 and 36 days. Seeds of the HS species were exposed to AD at both temperatures for 3, 9, 18, and 36 days (Supplementary Table 2). However, since the seeds of *A. theophrasti – 1 year* and *M. alcea – 1 year* were harvested after the experiments with exposure times up to 36 days at 35°C had already started, they were exposed to AD at 35°C for only 1, 3, 6, and 9 days (Supplementary Table 2).

For most species, four replicates were taken at each combination of exposure time and temperature. As seed survival is known to decrease with increasing exposure time (Westerman and Gerowitt, 2013), the number of seeds was increased with exposure time in order to optimize the discriminative power of the assay: Depending on the exposure time, 100, 200, or 300 seeds of a species were anaerobically digested per replicate (Supplementary Table 2).

#### Determination of Seed Viability

After AD, seeds were rinsed with water, transported to the laboratory in Rostock and processed within 5 h after removal from the reactor. Seed viability was determined by testing the germination and metabolic activity of all seeds that did not germinate within 21 days.

In detail, surface sterilized seeds were incubated on plates with “diaspore agar” (agar 13.0 g l^–1^, KNO_3_ 2.0 g l^–1^, gibberellic acid 0.5 g l^–1^, ampicillin 0.1 g l^–1^, streptomycin 0.1 g l^–1^, benzimidazole 0.02 g l^–1^) at 20/4°C day/night temperatures with a 16 h photoperiod. The number of germinated seeds on the plates was recorded at regular intervals during 21 days. A seed was considered germinated if the radical protruded at least 2 mm from the seed. The viability of all remaining non-germinated seeds was tested using 2,3,5-triphenyltetrazolium chloride (TTC) that indirectly determines the metabolic activity and thus viability of seed tissue cells (França-Neto and Krzyzanowski, 2019). To enable the TTC molecules to enter the seed, seed coats were carefully punctured with a needle or scalpel without inflicting injuries to the embryo (Elias et al., 2012). The punctured seeds were placed between two filter papers, soaked with 3 ml of 1.0% TTC solution and incubated in the dark at 35°C for 20–22 h. Based on the guidelines in the Tetrazolium Testing Handbook (Association of Official Seed Analysts, 2010) seeds were judged fully viable but dormant if the embryo - and endosperm, if relevant - was stained red. Embryos that were not stained (white), poorly stained (light pink), or lacked staining in areas critical for normal seedling development were classified as non-viable. Likewise, seeds whose embryo had rotted or which had already been degraded in the reactor and therefore could not be recovered (lost) also fell into the non-viable category. Ultimately, this test procedure provided the number of seeds that (1) germinated during the 21-day germination test after AD-treatment, (2) remained dormant but metabolically active, and (3) were not viable.

The same procedure was used to determine the germination and viability of control seeds that had not been exposed to AD (minimum of three replicates of 300 seeds per species, Supplementary Table 2). In preparation for the germination tests, the previously dry stored control seeds were exposed to a water-saturated atmosphere for 2 days in the dark.

### Statistical Analyses

#### Seed Viability Models

Both germinated and dormant seeds were viable after the different exposure times in mesophilic AD. For each replicate, the proportion of viable seeds, *V*, was calculated by dividing the cumulative number of germinated seeds after 21 in the germination test plus the number of dormant seeds by the total number of evaluated seeds (eq. 1).


(1) $$V=\frac{\sum \mathrm{germinated}\,\mathrm{seeds}+\sum \mathrm{dormant}\,\mathrm{seeds}}{\sum \mathrm{total}\,\mathrm{number}\,\mathrm{of}\,\mathrm{evaluated}\,\mathrm{seeds}}$$

With *V*, proportion of viable seeds observed in a replicate after a certain exposure time to AD; Σgerminated seeds, cumulative number of germinated seeds from the 21-day germination test; Σdormant seeds, number of seeds viable in tetrazolium testing after the germination test; Σtotal *numberofevaluatedseed*, total number of evaluated seeds in the replicate.

All statistical analyses were carried out using the software environment R (version 4.1.2) (R Core Team, 2021). Seed viability as a function of exposure time, *V(t)*, was modeled with a dose-response approach using the R-package “drc” (version 3.0.1, Ritz et al., 2015). The response variables, *V* or *V(t)*, mean the “observed *V*” of the samples or its values modeled over time. Log-logistic models with a lower limit of zero were fitted to the observed proportions of viable seeds (eq. 2, Supplementary Figure 2A). Models were fitted species-wise for both temperatures simultaneously by setting temperature as a grouping variable. The 35°C- and 42°C-models shared the upper horizontal asymptote, i.e., the maximum proportion of viable seeds, *V*_*max*_. The data type was “binomial” and the total number of evaluated seeds was set as weights. The model fit was evaluated both by a Chi^2^-test and visually (Supplementary Table 3). In case all or almost all seeds had lost viability even after the shortest exposure time to AD (1 day or 3 days), no model was fitted.


(2) $$V\,\left(t\right)=\frac{V_{m\,a\,x}}{1+e^{S\,L\,P\,\left(\log\left(t\right)-\text{log}\,\left(M\,I\,T\right)\right)}}$$

With *V(t)*, proportion of viable seeds as a function of the time of exposure in AD (*t*); *V*_*max*_, maximum proportion of viable seeds (upper asymptote); *SLP*, parameter proportional to the slope of *V(t)* in the inflection point; *MIT* (median inactivation time), the time after which *V(t)* reaches 50% of V*_*max*_*.

Due to viability increases in AD, the log-logistic models did not provide a good fit for *M. sylvestris*, *M. albus*, and *M. officinalis.* To improve their fit, one parameter was added to the log-logistic models using the Brain-Cousens modification (Ritz and Streibig, 2016, eq. 3). In these models, the lag-phase at the beginning of exposure is replaced by a sigmoid curve describing hormesis, and thus, turning a monotonically decreasing dose-response relationship into a biphasic one (Cedergreen et al., 2005; Kendig et al., 2010; Supplementary Figure 2B).


(3) $$V\,\left(t\right)=\frac{V_{m\,a\,x}+H}{1+e^{S\,L\,P\,\left(\log\left(t\right)-\text{log}\,\left(E\right)\right)}}$$

With *V(t)*, proportion of viable seeds as a function of the time of exposure in AD (*t*); *V*_*max*_, maximum proportion of viable seeds (upper asymptote); *H*, size of the hormesis effect, i.e., stimulation of viability at t close to zero; *SLP*, parameter changing the slope of the model curve; *E*, parameter shifting and stretching the model curve.

From the viability models the median inactivation times (*MITs*) and decimal reduction times (*DRTs)* were estimated, i.e., the times required to reduce viability to 50 or 10% of the initial viability, respectively (Supplementary Figure 2). If models could be fitted to the data obtained for both 35 and 42°C, parameter estimates, *MIT* and *DRT* were compared between the two temperatures species-wise using the “drc”-built-in functions *compParm* and *EDcomp* (Ritz and Streibig, 2016). The level of significance, α, was set to 0.05.

#### Seed-Killing Efficacy

To assess how much AD reduced seed viability, the viability models were used to estimate the mean *V* and standard error after 36 days for AD at both 35 and 42°C. Based on these data and the approach of Hahn et al. (2021), the seed-killing efficacies of 36 days in AD were calculated as follows (eq. 4):


(4) $$seed-killingefficacy\left(\%\right)=100\times \left(1-\frac{V\,\left(36\,d\,a\,y\,s\right)}{V\,\left(0\,d\,a\,y\,s\right)}\right)$$

#### Cumulative Germination and Dormant Seeds

In addition to total viability, its components, i.e., the proportions of germinated and dormant seeds, were analyzed individually. Cumulative germination after the 21-day germination test, *cG*, was modeled as a function of exposure time, *t*, similar to *V(t)*. Due to the skewness of the data, however, an asymmetric, three-parameter Weibull type 1 function with a lower limit of zero was chosen for most seed lots (eq. 5). The only exception to this was *A. theophrasti – 7 years* for which a log-logistic model including hormesis was used (compare eq. 3). The model fit was evaluated by a Chi^2^-test and visually (Supplementary Table 4).

From the germination models decimal reduction times for *cG* (*DRT(cG))* were estimated and compared to *DRT(V)* for 35°C and 42°C. In addition, the proportion of dormant seeds, *D*, was calculated from the difference between the models for *V(t)* and *cG(t).* Finally, the percentages of germinated, dormant, and inactive seeds were calculated in the untreated controls and after 36 days in AD at 35°C and 42°C, respectively.


(5) $$c\,G\,\left(t\right)=c\,G_{m\,a\,x}\,e^{-e^{S\,L\,P\,\left(I\,F\,T-\log\left(t\right)\right)}}$$

With *cG(t)*, proportion of cumulative germination at the end of the 21 days germination test, *cG*, as a function of the time of exposure in AD (*t*); *cGmax*, maximum proportion of *cG* (upper asymptote); *SLP*, parameter proportional to the slope of *cG(t)* in the inflection point; *IFT* (inflection time): the time after which the curve of *cG(t)* changes its flection.

## Results

### Seed Viability

#### Responses During Anaerobic Digestion

**FIGURE 2:**
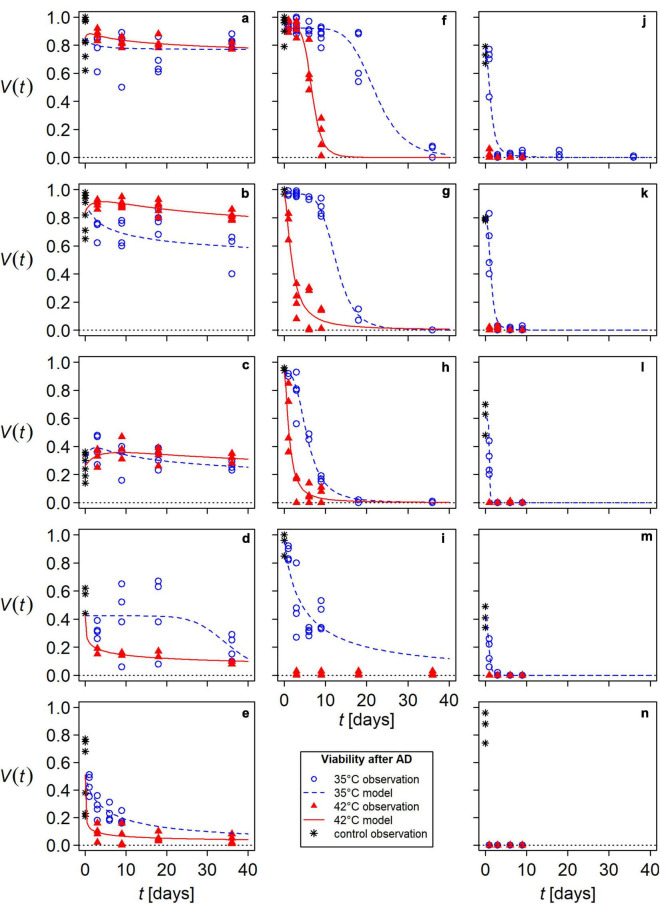
Modeled seed viability, *V*, of flowering wild plant species and tomato during anaerobic digestion (AD) in lab-scale reactors at 35°C (blue, dashed lines) and 42°C (red, solid lines). Symbols present observations, each containing a minimum of 100 seeds: asterisks for untreated controls, blue open circles for AD at 35°C and red filled triangles for AD at 42°C. **(a)** *Melilotus officinalis*, **(b)** *Melilotus albus*, **(c)** *Malva sylvestris*, **(d)** *Malva alcea – 2 years*, **(e)** *Malva alcea – 1 year*, **(f)** *Chenopodium album*, **(g)** tomato – PIERRE, **(h)** tomato – PAPRIKA, **(i)** *Abutilon theophrasti – 1 year*, **(j)** *Abutilon theophrasti – 7 years*, **(k)** *Daucus carota*, **(l)** *Cichorium intybus*, **(m)** *Echium vulgare*, **(n)** *Verbascum thapsus*. For better comparability, panels were arranged according to the species’ viability after 36 days of exposure to AD.

Viability responses to mesophilic AD varied among species and among seed lots of a species (Figure 2). The species most resistant to mesophilic AD were *M. officinalis*, *M. albus*, and *M. sylvestris* (Figures 2a–c and Table 1). The species whose seeds were inactivated most rapidly was *V. thapsus* (Figure 2n and Table 1).

**TABLE 1:** Estimated median inactivation times (MITs) and decimal reduction times (DRTs) of seeds of flowering wild plant species and tomato after anaerobic digestion (AD) at 35 or 42°C in lab-scale reactors, and corresponding seed-killing efficacy of AD.

	MIT [days]		DRT [days]		Seed-killing efficacy [%] of 36 days in	
	AD 35°C	AD 42°C	AD 35°C	AD 42°C	AD 35°C	AD 42°C
**HS species**						
*Abutilon theophrasti – 7 years*	1.3 *(0.06)*	<1	3.4 *(0.14)*	<1	100	100
*Abutilon theophrasti – 1 year*	4.7 *(0.24)*	<1	51.9 *(8.27)*	<3	86	99
*Malva alcea – 2 years*	35.4 *(0.65)*	1.7 *(1.36)*	46.9 *(6.29)*	>365	52	76
*Malva alcea – 1 year*	5.1 *(0.68)*	0.1 *(0.06)*	76.7 *(27.70)*	18.4 *(4.98)*	82	92
*Malva sylvestris*	>365	>365	>365	>365	0	−23
*Melilotus albus*	>365	>365	>365	>365	36	2
*Melilotus officinalis*	>365	>365	>365	>365	9	7
**NHS species**						
*Chenopodium album*	22.1 *(0.30)*	6.8 *(0.07)*	31.1 *(0.60)*	9.8 *(0.17)*	96	100
*Cichorium intybus*	1.0 *(0.02)*	<1	1.2 *(0.60)*	<1	100	100
*Daucus carota*	1.3 *(0.06)*	<1	2.7 *(0.13)*	<1	100	100
*Echium vulgare*	0.9 *(0.06)*	<1	1.6 *(0.13)*	<1	100	100
*Verbascum thapsus*	<1	<1	<1	<1	100	100
tomato – PAPRIKA	5.6 *(0.18)*	1.3 *(0.07)*	11.5 *(0.37)*	4.6 *(0.22)*	100	100
tomato – PIERRE	12.6 *(0.20)*	1.9 *(0.09)*	18.4 *(0.51)*	7.3 *(0.33)*	100	100

*Species are grouped according to their potential to exhibit hardseededness (HS) in their seeds or not (NHS). Standard errors of the mean are given in parentheses. Standard errors were not calculated when models could not be fitted because seeds were completely inactivated even after the shortest exposure time (“<1” or “<3”), or when estimated values exceeded 1 year (365 days, “>365”).*

For all species except *M. sylvestris*, *M. albus*, and *M. officinalis*, seed viability continuously decreased with increasing exposure time in the lab-scale reactors. A more or less pronounced lag-phase was followed by an exponential decrease in viability, which led either to complete seed inactivation or a plateau. If a plateau was reached, it was lower at 42°C than at 35°C. In addition, the viability curves dropped more steeply in AD at 42°C than at 35°C (Figures 2d–n).

The viability curves of the particularly AD-resistant HS species *M. officinalis*, *M. albus*, and *M. sylvestris* were biphasic: modeled viability initially increased during the first days of AD (Figures 2a–c). This means that the observed viability of samples treated with AD was higher than that of the untreated controls. This observed increase was most pronounced in *M. sylvestris*, with some individual values exceeding the maximum of the controls. Modeled viability of *M. sylvestris* reached its maximum of 149 and 136% of untreated controls, respectively, in AD at 35°C after 2.5 days and in AD at 42°C after 8.9 days. Subsequently, viability decreased, but after 36 days in AD at 35°C, it was as high as in the untreated controls and even 23% higher at 42°C (Figure 2c). In the two *Melilotus* species, the increase in viability was lower than in *M. sylvestris*, remaining below the maximum value of the untreated controls. Furthermore, the increase occurred after a shorter time and was stronger in AD at 42°C than in AD at 35°C. Maximum viability values at 42°C were 108% for *M. albus* (4.3 days) and 103% for *M. officinalis* (0.9 days) (Figures 2a,b). In addition, *M. albus* tended to lose viability more rapidly in AD at 35°C than at 42°C (Figure 2b). In tendency, this was also observed in *M. officinalis* and *M. sylvestris* (Figures 2a,c).

#### Seed-Killing Efficacies

Averaged over all species and both temperatures, the mean seed-killing efficacy (*SKE*) of 36 days in mesophilic AD was 76 ± 40% (*n* = 28). However, the values for NHS species, particularly AD-resistant HS species, and the remaining HS species (*A. theophrasti* and *M. alcea*) differed greatly (Table 1). According to the viability models, NHS species were completely inactivated after 36 days. The only exception was *C. album*, of which 4 ± 1% of the seeds remained viable after 36 days in AD at 35°C (Table 1). Of the NHS species, only *Chenopodium album* and tomato were able to survive AD beyond 9 days of exposure at both temperatures (Figures 2f–h). *Daucus carota* seeds were only 1% viable after 9 days when anaerobically digested at 35°C (Figure 2k). Survival of all other NHS species was poorer: *C. intybus*, *E. vulgare*, and *V. thapsus*, survived less than 3 days at 35°C and less than 1 day at 42°C (Figures 2l–n). The mean *SKE* on the HS species *A. theophrasti* and *M. alcea* was 86 ± 16% (*n* = 8). The lowest mean *SKE* was determined for the particularly AD-resistant HS species. It was only 5 ± 19% (*n* = 6) with a range of −23 to 36% (Table 1).

#### Inactivation Times

Inactivation times showed a wide range, with the longest determined for *M. officinalis*, *M. albus*, and *M. sylvestris*. To inactivate 50% (*MIT*) or 90% (*DRT*) of their originally viable seeds, it was estimated that more than 1 year would have been required. The *DRT*s for the NHS representatives from the wildflower biogas mixture ranged from only a few hours to 2.7 ± 0.13 days (*D. carota*). The inactivation times for the other species fell between these values. With 31.1 ± 0.60 days and 9.8 ± 0.17 days at 35°C and 42°C, respectively, *DRT* values for *C. album* were in a similar range to those of the HS species, *A. theophrasti* (Table 1).

For most species, inactivation times were shorter in AD at 42°C than in AD at 35°C. Exceptions were three particularly AD-resistant species and *M. alcea – 2 years* (Table 1). In addition, inactivation times differed between the seed lots of a species. The tomato variety PAPRIKA was inactivated faster than the variety PIERRE (Figures 2h,g and Table 1). The batch *A. theophrasti – 1 year* had a 15-times longer *DRT* at 35°C than the 7-year old batch and even a few viable seeds remaining after 36 days in AD at 42°C (Figures 2i,j and Table 1). The difference between the two seed lots of *M. alcea* was most apparent when comparing their response to AD at 35°C: inactivation in *M. alcea – 2 years* was preceded by a 21-day lag phase, whereas it started immediately in *M. alcea – 1 year* (Figures 2d,e). As a result, it took considerably longer to reduce the viability of the older *M. alcea* by 50% than the younger one (*MIT*: 35 days instead of 5 days), but it took less time to reduce it by 90% (*DRT*: 47 days instead of 77 days) (Table 1).

### Germinable and Dormant Seeds

#### Before Anaerobic Digestion

**FIGURE 3:**
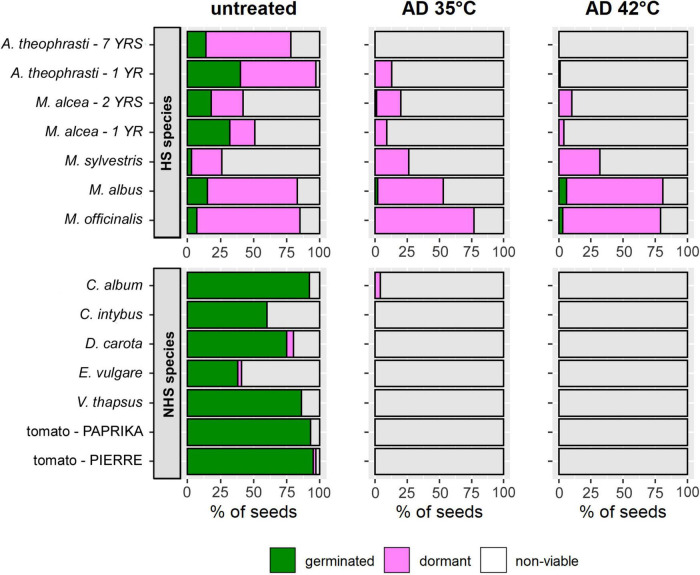
Mean percentages of germinated (green), dormant (pink), and non-viable seeds (gray) in batches of flowering wild plant species and tomato that were either untreated (left) or exposed to AD at 35°C (center) or 42°C (right) for 36 days. The top row shows the values for species with hardseededness (HS), the bottom row those for non-hardseeded (NHS) species. The percentage of germinated seeds equals the cumulative germination (*cG*) after completion of the 21-day germination test after AD-treatment. Values were predicted from models for *V(t)* and *cG(t)*.

Untreated controls of HS and NHS species differed in the contribution of germinable and dormant seeds to viability (Figure 3, left). In NHS species, an average of 98 ± 3% (*n* = 7) of all viable seeds germinated. Dormant seeds occurred only in *D. carota*, *E. vulgare*, and tomato – *PIERRE* (Figure 3, left, bottom). In contrast, in the untreated samples of HS species, only 29 ± 20% (*n* = 7) of the viable seeds had germinated, while the rest remained dormant (Figure 3, left, top).

#### During Anaerobic Digestion

**FIGURE 4:**
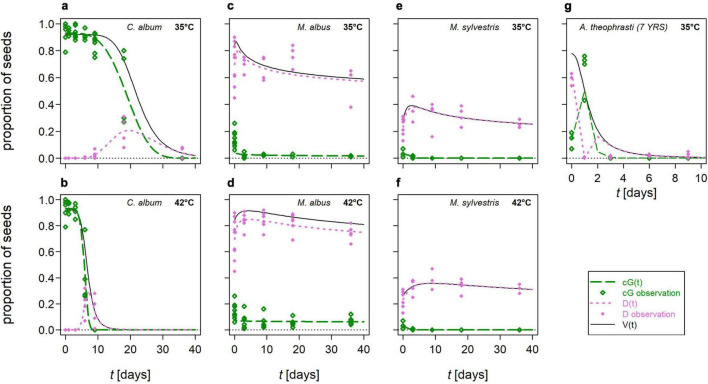
Modeled proportions of germinated (cumulative germination, *cG*, green long-dashed lines) and dormant seeds (*D*, pink dotted lines) out of all viable seeds (*V*, black solid lines, compare Figure 3) of selected flowering wild plant species after mesophilic, anaerobic digestion. *D(t)* was calculated from the difference between the models for *V(t)* and *cG(t)*. Symbols represent observations, each based on a minimum of 100 seeds: green open diamonds for *cG* and pink closed diamonds for *D*. **(a,b)** *Chenopodium album* at 35 and 42°C, respectively; **(c,d)** *Melilotus albus* at 35 and 42°C, respectively; **(e,f)** *Malva sylvestris* at 35 and 42°C, respectively; **(g)** *Abutilon theophrasti – 7 years* at 35°C. Please note the different scaling of the *x*-axis in **(g)**.

The contribution of germinable (cumulative germination, *cG*) and dormant seeds, *D*, to (total) viability, *V*, differed between HS and NHS species during the different exposure periods in AD (Figure 4). In NHS species, the majority of viable seeds germinated, and the loss of *V* corresponded to an approximately equal loss of *cG* (e.g., *C. album* in Figures 4a,b). This nearly simultaneous loss of *cG* and *V* was reflected in the fact that *DRTs* of *cG* were at most 5 days shorter than those of *V* (Supplementary Table 5). In all HS species except *A. theophrasti*, most viable seeds remained dormant during exposure to mesophilic AD. The viability curve paralleled that of the proportion of dormant seeds (e.g., *M. albus* in Figures 4c,d, *M. sylvestris* in Figures 4e,f). The time interval between 90% loss of *cG* and *V* was not days as in NHS species, but weeks or months (Supplementary Table 5).

Only in *A. theophrasti cG* peaked during the first 3 days of exposure and not in the untreated controls as in the other species. For instance, in the *A. theophrasti – 7 years* seed lot, after 1 day in AD at 35°C, *cG* was five-times higher than in the untreated control. This peak of germinable seeds was paralleled by a local minimum of dormant seeds. However, *D* increased again as soon as the *cG-*peak exceeded its maximum (Figure 4g and Supplementary Figure 3J).

In four species, namely *A. theophrasti*, *C. album*, *D. carota* and tomato, a peak of dormant seeds was observed after *cG* and *V* began to decline. In the most extreme case, *C. album*, these *D*-peaks could account for up to one-third of *V* and last up to 30 days (Figure 4a). After longer exposure times, the curves of *D* and *V* finally coincided. Thus, the proportion of *D*, accounted for the sum of all seeds that were still viable after AD.

#### After 36 Days in Anaerobic Digestion

Seeds that were still viable after 36 days in mesophilic AD were almost exclusively dormant. This was also the case for the only surviving NHS species, *C. album* (Figure 3, middle + right). Small proportions of germinated seeds were only found in *M. alcea*, *M. albus* and *M. officinalis* (Figure 3, middle + right, top). The highest percentages of germinated seeds were observed for *M. albus* and *M. officinalis*: 0.06 and 0.03, respectively, after 36 days of AD at 42°C (Figure 3, right, top). Furthermore, only in the *Melilotus* species did more seeds germinate after AD at 42°C than at 35°C. Finally, only in *Melilotus* species were bare, i.e., seed coat-less, embryos frequently found in the sample bags in addition to seeds that were recognizable as viable or non-viable. When some of the bare embryos were subjected to a germination test, it became apparent that they were able to develop and grow.

## Discussion

Mesophilic AD affected seed viability of the species studied to varying degrees, and none of the potentially modulating factors had a consistent effect. We rejected the hypotheses: mesophilic AD, reduced seed viability of one of the HS species more than that of two of the NHS species (hypothesis 1). Seed viability of three species did not decrease markedly more at the higher incubation temperature than at the lower one (hypothesis 2). The observed seed viability of three species increased when exposure time increased up to a certain point (hypothesis 3). Instead, we report a more complex response of the wildflower seeds studied: more factors than HS affected AD-resistance (see section “Anaerobic Digestion-Resistance of Hardseeded and Non-hardseeded Species”). Nevertheless, some of the HS species were particularly AD-resistant (see section “Particularly Anaerobic Digestion-Resistant Hardseeded Species”). Furthermore, responses were dependent on both germinable and dormant seeds (see section “Diversity of Viability Responses”). These findings have implications for the use of flowering wild plants as biogas feedstocks (see section “Flowering Wild Plants as Biogas Feedstocks”).

### Anaerobic Digestion-Resistance of Hardseeded and Non-hardseeded Species

In this study, seed-killing efficacies tended to be lower and inactivation times longer in HS species than in NHS species. However, inter- and intraspecific variation was high. Considering together viability curves, seed-killing efficacies, and inactivation times, it appears that the HS and the NHS species studied overlapped in AD-resistance potential, with NHS species tending to be at the “low” end of the scale and HS species at the “high” end. The HS species *A. theophrasti* and the NHS species *C. album* and tomato mainly caused this overlap. The most AD-resistant NHS species in this study was *C. album*, and its maximum survival times were in the upper ranges of values from comparable studies (Schrade et al., 2003; Katovich et al., 2004; Leonhardt et al., 2010; Westerman et al., 2012a,b; Johansen et al., 2013; Oechsner et al., 2018; Zhou et al., 2020). The same, although less pronounced, was true for tomato (Engeli et al., 1993; Lorenz et al., 2001; Schrade et al., 2003; Marcinisyn et al., 2004; Westerik and Kleizen, 2006; Strauß et al., 2012; Westerman et al., 2012a,b; Baute et al., 2016). Whether this was due to seed lot characteristics or different AD-conditions cannot be determined. However, several other NHS species survived mesophilic AD treatments for more than 1 week: *Amaranthus retroflexus* L. (common tumbleweed, Šarapatka et al., 1993; Katovich et al., 2004), *Digitaria sanguinalis* (L.) SCOP. (purple crabgrass (Engeli et al., 1993), *Echinochloa crus-galli* (L.) P. BEAUV (common barnyard grass, Šarapatka et al., 1993), *Panicum dichotomiflorum* MICHX. (autumn millet, Jeyanayagam and Collins, 1984), *Rumex obtusifolius* L. (broadleaf dock, Engeli et al., 1993; Šarapatka et al., 1993) and *Sorghum halepense* (L.) PERS. (Jeyanayagam and Collins, 1984). The survival of these NHS species supports the suggestion that HS is not the only mechanism by which seeds can gain resistance to AD (Westerman and Gerowitt, 2013). In the case of *C. album* and tomato a physically hard (but not HS) or thick seed coat has been discussed as the basis of their AD resistance (Blackshaw and Rode, 1991; Strauß et al., 2012; Aper et al., 2014). The protection provided by this hard seed coat would be lost during AD by microbial degradation processes; first slowly (lag-phase) and then increasingly rapidly. However, it is unclear whether this mechanism alone is sufficient to explain the remarkably high AD-resistance of *C. album* and tomato in this study.

Resistance differences within HS species were likely related to the fact that both the degree and depth of HS varies among species and seed lots (Baskin and Baskin, 2004). A good example of this is the 7-year-old seeds of *A. theophrasti* used in this study, which have already been tested in two other studies. Two years after harvest, about half of *A. theophrasti* seeds survived 30 days of AD in lab-scale batch reactors at 37°C (Westerman et al., 2012b) and all seeds were inactivated after 9 days of AD in commercial fermenters at 41°C (Westerman et al., 2012a). Now 7 years old, this seed lot was completely inactivated after 36 days in AD at both 35 and 42°C, and *DRT* was shorter at 42°C (<1 day) than at 41°C (2 days, Westerman et al., 2012a). Since other differences can be excluded here, AD-resistance must have decreased due to seed aging during storage (Sano et al., 2016). Aging and loss of AD-resistance appeared to be associated with a reduction in the depth of HS, as the degree of HS was higher at older ages (82% of “hard” seeds in the seed lot) than at younger ages (about 65%, Westerman et al., 2012b). A lower HS depth would also be consistent with the observation that the older seed lot germinated more and was less AD-resistant than the 1-year-old *A. theophrasti* lot used in this study, which had an even lower degree of HS (59%). However, unlike the first comparison, these two seed lots did not differ only in storage duration. This implies that differences in their AD-resistance could also be due to other factors leading to variations in HS, such as genetic differences, weather and site conditions, seed maturity, endogenous dormancy rhythms, and conditions of storage (Rolston, 1978; Baskin and Baskin, 1998; Hilhorst, 1998; Hay and Probert, 2013; Jaganathan et al., 2016). In this context, Westerman et al. (2012b) noted that genetic factors or environmental conditions during seed filling and maturation could be responsible for the difference in survival probability of mesophilic AD between populations of *A. theophrasti* harvested in different years and/or locations. Similarly, the higher AD-resistance of the older *M. alcea* batch in this study can be explained by the fact that its seeds were more mature and therefore had greater depth (Goldberg et al., 1994) and higher degree of HS (58% versus 38%) than the younger batch.

The influence of different seed lots on AD-resistance is not limited to HS species. Different seed lots of NHS species responded differently to AD as well (Traveset, 1998; Westerman et al., 2012b; Zhou et al., 2020). Consequently, AD-resistance seems determined by both species-specific traits and characteristics of the respective seed lots. This raises the question of (1) what factors affecting seed lot quality lead to differences in AD-resistance and (2) how large the differences between seed lots are compared to the differences between species. In addition, the relative importance of seed traits and AD-conditions on seed-killing efficacy would need to be determined.

### Particularly Anaerobic Digestion-Resistant Hardseeded Species

Compared to the other species examined in this study, the HS species *M. officinalis*, *M. albus*, and *M. sylvestris* were particularly resistant to mesophilic AD, tended to lose viability more slowly and to germinate more after AD at 42°C than at 35°C, and showed an initial increase in observed viability.

The seed-killing efficacy of AD on *M. officinalis*, *M. albus* and *M. sylvestris* was very low compared to other members of the Fabaceae and Malvaceae. The four other Fabaceae species studied to date, namely *Glycine max* (L.) MERR. (soybean), *Lupinus polyphyllus* LINDLEY (garden lupin), *Trifolium pratense* L. (red clover) and *Vicia tetrasperma* (L.) Schreb. (smooth vetch) had lost substantially more than 9% – 36% viability when exposed to similar AD-conditions (35–38°C, 7–30 days) (Leonhardt et al., 2010; Strauß et al., 2012; Westerman et al., 2012b; Hassani et al., 2021). Among the three members of the genus *Malva* studied, seed-killing efficacy was higher in *M. alcea* (this study) and *Malva neglecta* Wallr. (dwarf mallow) (Westerman et al., 2012a,b) than in *M. sylvestris*. It is particularly noteworthy that after 36 days of AD, the measured values for seed viabilities of *M. albus* and *M. officinalis* were still in the range of the values of the untreated controls. Values of *M. sylvestris* were even higher than in the controls, resulting in negative SKEs. The high AD-resistance distinguishes *M. officinalis*, *M. albus* and *M. sylvestris* from all other species.

The second unique characteristic of the particularly AD-resistant HS species was their tendency to lose viability faster and more severely at 35°C than at 42°C. Additionally, the range of responses was wider at 35°C. This was in contrast to all other species studied to date. However, it must be put into perspective that (a) this response was prominent only in *M. albus* and (b) other Fabaceae and Malvaceae were each exposed to only one temperature in previous studies (Katovich et al., 2004; Leonhardt et al., 2010; Strauß et al., 2012; Westerman et al., 2012a,b; Hassani et al., 2021). Some of our observations in *Melilotus* sp. suggest that fatal germination has played a role in the unexpected and rather counterintuitive response to temperature increase. First, we found bare but germinable embryos of *M. albus* and *M. officinalis* in the sample bags, indicating that AD triggered germination in these species. As a result, seeds would die due to thermosensitivity (Westerman and Gerowitt, 2013) and degrade, unless germination was triggered just before samples were removed from the reactor. Second, fewer *Melilotus* seeds germinated after AD at 35°C than after AD at 42°C, which may indicate that AD at 35°C caused more seeds to germinate already in the reactor and then die. In this way, viability would decline more at 35°C than at 42°C. It also cannot be ruled out, however, that the tendency of higher survival probability at the higher temperature was related to HS or the increase in observed viability.

Exclusively in *M. officinalis*, *M. albus*, and *M. sylvestris*, there was no lag-phase at the beginning of the AD-treatment, but an increase in observed seed viability. The resulting biphasic viability curves extend the spectrum of known responses to AD because, previously, such increases in seed viability in AD have only been reported for NHS species (Schrade et al., 2003; Westerik and Kleizen, 2006; Leonhardt et al., 2010; Baute et al., 2016; Zhou et al., 2020). In NHS species, the initial viability increase in AD was associated with the breaking of dormancy and initiation of germination (Westerik and Kleizen, 2006; Leonhardt et al., 2010; Zhou et al., 2020). However, in these three studies, germinability was equated with seed viability, which unfortunately does not allow conclusions to be drawn about total viability, i.e., germinating plus dormant seeds. Combining germination and TTC tests, we found that the increase in observed viability of the highly AD-resistant HS species was not due to an increase in germinating seeds, but to that of dormant seeds. This means that the total observed viability of the seed lot increased compared to the untreated control. The only study we know of that attempts to explain a similar result examined *Heracleum mantegazzianum* SOMMIER & LEVIER (giant hogweed) in a water bath at 35°C (Tanke et al., 2019). The authors attributed the increase in seed viability after 12 h to insufficient hydration of previously dry-stored control seeds. Hence, the TTC assay failed to capture the full respiratory potential of the seeds under these conditions (Elias et al., 2012; Miller, 2014). In other words, the observed viability increase was assumed to be an artifact. However, the controls in our study were well hydrated due to the pretreatment and germination test before the TTC test. Moreover, unlike the NHS species *H. mantegazzianum*, the HS species *M. officinalis*, *M. albus* and *M. sylvestris* in this study were not inactivated shortly after the viability peak, but their observed viability remained at a high level. Therefore, we hypothesize that the increase in observed viability was due to AD-induced metabolic stimulation of seeds whose metabolic activity was not detectable by TTC staining before AD. If this was the case, the increase in seed viability would be a form of hormesis, i.e., a dose-response phenomenon in which low doses of a stressor - here, brief exposure to AD - have a stimulatory effect, whereas high doses cause inhibition (e.g., Calabrese and Baldwin, 2002; Mattson, 2008; Kendig et al., 2010). To date, hormesis has been demonstrated in over 15,000 experimental studies (Kozumbo and Calabrese, 2019). And biogas reactor conditions, e.g., heat, enzymes, amino and organic acids, alcohols, hydrogen sulfide, ammonia, and cyanides (Westerman and Gerowitt, 2013), resemble stressors known to induce hormesis (e.g., Calabrese et al., 2007). The assumed stimulation by AD would have to activate processes that can repair (oxidative) damage to DNA, membranes, proteins, etc., that occurred before the AD treatment, i.e., during maturation and storage of the seed. Examples of such processes include activation of DNA repair mechanisms and antioxidant (enzyme) systems, expression of growth factors, anti-apoptotic proteins and heat shock proteins, and de-novo synthesis of cellular components. These processes occur in established, conserved hormetic pathways (Calabrese et al., 2007; Mattson, 2008) and enhance seed vigor during pre-germinative metabolic events utilized in seed priming (Paparella et al., 2015; Lutts et al., 2016). However, the observed viability increases in *M. officinalis*, *M. albus* and *M. sylvestris* may also be due to limitations of TTC testing that were detectable only in these three species. It is conceivable that the metabolic activity of (AD-)microorganisms attached to the seeds resulted in an apparent increase in seed viability. With the same result, AD may have facilitated TTC uptake in these HS species. Furthermore, TTC tests are invasive, so the viability of the AD-treated samples in the untreated state may have varied more than that of our controls, even though all samples were from the same seed lot. Then, there is the question of the extent to which the timing and mechanisms of an increase in viability differ between HS and NHS species. The HS species in this study took between 6 hours and 9 days longer to reach the viability maximum than NHS species in other studies (Schrade et al., 2003; Westerik and Kleizen, 2006; Leonhardt et al., 2010; Baute et al., 2016; Zhou et al., 2020). Thus, the shortest sampling interval of 24 h could have been too long to detect viability increases in all species studied here. Finally, given the particular AD-resistance of these HS species, it is of particular interest to determine whether or not the hypothesized metabolic stimulation is due to processes that require imbibition. This aspect is important because repair processes are normally associated with (partial) rehydration of seeds (Weitbrecht et al., 2011; Powell and Matthews, 2012; Paparella et al., 2015; Sano et al., 2016), but the protective effect of HS against AD is irreversibly lost once the water impermeability of the seed coat is broken and seeds absorb water (Westerman and Gerowitt, 2013). However, it is also possible that metabolic stimulation was triggered by factors other than rehydration, because unimbibed, dry seeds can have low-level metabolic activity (e.g., Weitbrecht et al., 2011; Sano et al., 2016) and even priming effects are not necessarily related to seed imbibition (Lutts et al., 2016). In summary, to clarify whether hormesis can be triggered by AD in seeds, future studies should include measurements with a higher resolution in the range of the increase in observed viability and be complemented by molecular analyses.

### Diversity of Viability Responses

Observed responses during mesophilic AD ranged from complete inactivation to viability increases. Furthermore, responses differed between germinable and dormant seeds. Germinability was lost faster than viability in all species, indicating that mainly dormant seeds survived AD. In contrast to the NHS species, the HS species lost their germinability very quickly compared to their viability. And at the end of the measurement period, almost all of their surviving seeds were active but not germinating, thus, most probably physically dormant. These observations confirm that HS makes survival in AD more likely (Leonhardt et al., 2010; Westerman et al., 2012b; Jaganathan et al., 2016; Hassani et al., 2021). It remains unclear to what extent, however, as the HS species had lost so little viability at the end of the measurements. It appeared as if the remaining hard seeds could survive AD for even longer periods (viability plateau). The exceptionally long estimated inactivation times for the particularly AD-resistant HS species reflected this. However, they are unlikely to survive longer than a year in mesophilic AD, as the most resistant species studied to date survived only about 30 days (e.g., Jeyanayagam and Collins, 1984; Westerman et al., 2012b), and up to 155 days in extreme cases (Hassani et al., 2021). Moreover, seeds can be inactivated in AD despite intact physical dormancy (Westerman et al., 2012b) and viability plateaus can end quite abruptly, as observed in *M. alcea – 2 years* (Figure 2d). For these reasons, a realistic assessment of AD-resistance in HS species requires that measurements continue until both the dormant and non-dormant seeds are fully inactivated.

A special case with regard to its responses was *Abutilon theophrasti*. First, it was the only HS species to be completely (7 years old seed lot) or nearly completely (1 year old) inactivated at the end of the measurement period. Possible reasons for this have already been discussed (see section Anaerobic Digestion-Resistance of Hardseededness and Non-hardseeded Species). Second, mesophilic AD stimulated germination in it, which has previously been observed only in NHS species (Schrade et al., 2003; Westerik and Kleizen, 2006; Leonhardt et al., 2010; Baute et al., 2016; Oechsner et al., 2018; Zhou et al., 2020). Third, it was the only HS species in which peaks of metabolically active seeds (*D*-peaks) occurred when cumulative germination decreased. Otherwise, these *D*-peaks occurred only in *C. album*, tomato, and *D. carota*.

It remains to be clarified how the *D*-peaks are to be evaluated in terms of seed viability. Based on our investigations, we can either assume that the *D*-peaks represented the parts of the seed lots no longer protected by the seed coat. These would have been damaged, losing their ability to germinate and retaining only some metabolic activity, which we would have mistakenly interpreted as dormancy. If we follow this line of reasoning, we would agree with Eckford et al. (2012). They assumed that AD-treated seeds of *Fallopia convolvulus* L. (wild buckwheat) with pink-stained and thus theoretically viable embryos were not capable of normal growth and development, because a TTC test does not measure the capacity for normal cell division, growth speed, or dormancy (Copeland, 1976; Miller, 2014). However, for AD-treatments of perennial biomass species and tomato Baute et al. (2016) found that Petri-dish germination with TTC staining resulted in similar viability estimates as glasshouse germination with cold-moisture stratification. The overestimation by TTC staining was only 5%. Moreover, a TTC test may well determine the number of seeds that would develop normal seedlings in a germination test when all available viability indicators are included in the evaluation (Elias et al., 2012). We did this by classifying only the red-stained, physically intact embryos as viable. Thus, the *D*-peaks could represent fully viable, in principle germinable but dormant seeds. This would mean that secondary dormancy was induced by AD. Possible triggers such as heat, high moisture and low oxygen levels (Hilhorst, 1998; Bentsink and Koornneef, 2008) occur in biogas reactors. Overud (2002) previously speculated that the low oxygen content was responsible for the induction of secondary dormancy in *Rumex crispus* L. (curled dock) in ensiling, another anaerobic fermentation process. This reasoning, together with the observation that *D*-peaks occurred only in those NHS species that were relatively resistant to AD, raises the question of whether dormancy mechanisms other than HS contribute to AD-resistance in seeds. Considering that chemical and biological processes contribute to seed inactivation in addition to temperature (Westerman and Gerowitt, 2013; Zhou et al., 2020), any dormancy-related defenses against microbial or chemical attack (e.g., Fuerst et al., 2011; Chen et al., 2018) could increase the probability of seed survival in AD.

In summary, the way in which species and seed lot traits impact seed viability responses to AD seems multifaceted. Future studies should include physiological and molecular aspects in order to develop a more mechanistic view that allows to better predict seed viability in AD.

### Flowering Wild Plants as Biogas Feedstocks

Plant seeds that survive AD in a commercial biogas plant can get particularly widely distributed because the digestate is also traded between farms. In addition, repeated AD of seed-bearing biomass and its fertilization with digestate containing (viable) seeds can lead to the selection for AD-resistant biotypes (Westerman and Gerowitt, 2013). Under these conditions, a few surviving seeds would be sufficient for a new weed flora to emerge. Our finding that a portion of all tested HS species was still viable after 36 days of mesophilic AD suggests that these risks exist when using these species or the flowering mixture as a biogas feedstock. This is especially true for mesophilic biogas plants, which operate with shorter hydraulic retention times (HRTs) than in this study. In Germany, for example, 15% use HRTs between 15 and 35 days (vTI, 2009). In addition, approximately 1–10% of the freshly fed feedstock passes through the reactor after only 6–24 h (Turner et al., 1983; Baier et al., 2010; Eckford et al., 2012). Especially complete mix, one-stage reactors suffer from this short-circuiting (Ward et al., 2008). After such shortened retention times in AD, seeds that are either not yet inactivated or possibly even activated (hormesis, germination stimulation) can enter the digestate. Consequently, it would be safest to exclude HS species in the composition of biogas mixtures - and as a biogas feedstock in principle.

Excluding HS (wild plant) species as biogas feedstock, however, would mean losing their socio-ecological benefits (e.g., Kuhn and Vollrath, 2010; von Cossel, 2020), e.g., the nitrogen fixation of Fabaceae or the attractive flowering offer of Malvaceae. Furthermore, it is still unclear whether the risk of spread found in experiments applies in agricultural practice. In most commercial biogas plants, the proportion of surviving seeds is likely to be lower because the average HRT is longer than that tested, e.g., 91 days in Germany (vTI, 2009). The other steps in the biogas production chain can contribute to seed inactivation as well, namely cultivation, harvest, ensiling, storage, pre-treatment and digestate storage (Fröschle et al., 2015). On farms, biomass is usually ensiled prior to AD. In general, ensiling reduced the viability (e.g., Aper et al., 2014; Simard and Lambert-Beaudet, 2016; Piltz et al., 2017), including that of seeds from the flowering wild plant mixture (Hahn et al., 2021). When the same seed lot was both ensiled and anaerobically digested in biogas reactors (Westerman et al., 2012b) or in the rumen of cattle (Blackshaw and Rode, 1991; Mayer et al., 2000; Stanton et al., 2012; Aper et al., 2014; Piltz et al., 2017), seed-killing efficacy was mostly higher in ensiling than in AD. However, there was considerable variation between and within species. The same picture emerged when comparing the seed-killing efficacies of 36 days in mesophilic AD (Table 1) with those obtained for 8 months of ensiling by Hahn et al. (2021). The same seed lots of *C. album*, *C. intybus*, *V. thapsus*, *A. theophrasti – 7 years*, *M. alcea – 2 years*, *M. albus* and *M. officinalis* were used in both studies. In NHS species and *M. officinalis*, a greater proportion of seeds was killed by ensiling than by AD. In contrast, ensiling was less effective on *A. theophrasti – 7 years* and *M. alcea – 2 years*, inactivating only 5 and 23%, respectively, while AD killed 100% and about 60%, respectively. Consistent with this, Westerman et al. (2012b) found that their batch of *A. theophrasti* and the species *Malva neglecta* were more resistant to ensiling than to AD. Piltz et al. (2017), studying *Malva parviflora* L. (small-flowered mallow) and referring to *Malva pusilla* SM. (small mallow) studied by Blackshaw and Rode (1991), even suggested that resistance to ensiling may be characteristic of the Malvaceae genus. Finally, for *M. albus*, our results showed that seed-killing efficacy did not differ per se between the processes of ensiling and AD, but depended on the respective conditions: AD at 35°C killed 36% of *M. albus* seeds, ensiling killed 23% and AD at 42°C killed only 2% (Table 1; Hahn et al., 2021). Regarding a combined seed-killing efficacy of ensiling and AD, the few available sources indicate that it is about the same as that of the individual processes, but tends to kill more seeds (Blackshaw and Rode, 1991; Stanton et al., 2012; Westerman et al., 2012b; Piltz et al., 2017).

We conclude that it is not necessary to categorically exclude HS species to avoid unwanted seed spread when mixtures of flowering wild plant species are used as biogas feedstock. Rather, we recommend that conditions during AD be designed to increase the seed-killing efficacy. This can be achieved, for example, by sufficiently long HRTs in the biogas reactor without short-circuiting. Furthermore, HS species whose seeds are easy to inactivate should be identified. Considering that AD is only one step within the biogas process chain, seed-killing efficacy of AD may have been underestimated in our experiments conducted with mature, vigorous, dry-stored seed, because the seed in practice may be immature and damaged by upstream treatments, making it more susceptible to AD (Westerman and Gerowitt, 2013; Zhou et al., 2020). Therefore, the influence of cultivation parameters on seed quality, e.g., harvest timing, and the influence of feedstock storage conditions, pre-treatment and digestate processing technologies (Monfet et al., 2018; Alsanius et al., 2021) on seed viability remain to be explored. And even though our reactors were comparable to a full-scale fermenter in terms of operating parameters (Heiermann and Plogsties, 2018), our laboratory results require validation on the practical scale. Finally, seed establishment studies under field conditions are needed to realistically assess the dispersal risk of seeds that survive AD.

## Data Availability Statement

The raw data supporting the conclusions of this article will be made available by the authors, without undue reservation, to any qualified researcher on request.

## Author Contributions

JH organized the project, designed the study, analyzed the viability of the seeds, and drafted and edited the manuscript. PW analyzed the viability of the seeds and edited the manuscript in coordination with JH. FM contributed to performance and description of the statistical analyses in coordination with JH. MH administrated the operation of the lab-scale reactors and edited the manuscript in coordination with JH. BG led the project and contributed to the conception and design of the study. All authors contributed to manuscript revision, read, and approved the submitted version.

## Conflict of Interest

The authors declare that the research was conducted in the absence of any commercial or financial relationships that could be construed as a potential conflict of interest.

## Publisher’s Note

All claims expressed in this article are solely those of the authors and do not necessarily represent those of their affiliated organizations, or those of the publisher, the editors and the reviewers. Any product that may be evaluated in this article, or claim that may be made by its manufacturer, is not guaranteed or endorsed by the publisher.

## Footnotes

1
saaten-zeller.de

2
appelswilde.de

3
culinaris-saatgut.de

4
https://www.bingenheimersaatgut.de/de
