# Supplementary material for: Viability of Wildflower Seeds After Mesophilic Anaerobic Digestion in Lab-Scale Biogas Reactors
Source: Front Plant Sci. 2022 Jul 14;13:942346. doi: 10.3389/fpls.2022.942346 (PMC9337220; doi:10.3389/fpls.2022.942346)
Supplement: Supplementary file 1 [file Data_Sheet_1.docx]

Supplementary Data: Process control of lab-scale biogas reactors

A total of 12 continuously stirred laboratory-scale reactors were operated for the anaerobic digestion (AD) experiments (**Table S1**). The anaerobic digestion in the first reactors was initiated with a start-up phase on 29.01.2015. Feeding was carried out manually on six days per week. After reaching the intended organic loading rate (ORL = 3 g_VS_ l^-1^ d^-1^) and a stabilization phase, the insertion of bags containing wildflower plant seeds started on 10.03.2015. Although the reactors were opened frequently, operation was stable according to the monitored time courses of biogas yield, methane content and further process parameters (total solids, volatile solids, pH, ammonium nitrogen, total nitrogen, volatile fatty acids, pH) (**Table S1**, **Figure S1a – S1d**). Fluctuations in gas production resulted mainly from non-feeding on Sundays

Table S1. Chemical characteristics of the reactor-specific process fluid during the monitored time periods.

| **Reactor** | **T [°C]** | **n** |  | **TS  [% FM]** | **VS  [% DM]** | **pH** | **NH_4_-N  [g^.^l^-1^]** | **N_tot_  [g^.^l^-1^]** | **AA  [g^.^l^-1^]** | **PA  [g^.^l^-1^]** | **VFA  [g^.^l^-1^]** |
| --- | --- | --- | --- | --- | --- | --- | --- | --- | --- | --- | --- |
| 1 |  | 11 | mean | 7.6 | 79.3 | 7.5 | 1.6 | 4 | 0.35 | 0.07 | 0.41 |
|  | 35 |  | range | (6.8-8.6) | (77.0-81.7) | (7.3-7.7) | (1.4-1.8) | (3.4-4.8) | (0.16-0.49) | (0.00-0.33) | (0.18-0.73) |
|  |  |  | sd | 0.6 | 1.4 | 0.1 | 0.2 | 0.3 | 0.09 | 0.1 | 0.15 |
| 2 |  | 11 | mean | 7.7 | 79.5 | 7.5 | 1.6 | 4 | 0.37 | 0.06 | 0.42 |
|  | 35 |  | range | (6.9-8.4) | (76.9-81.8) | (7.4-7.6) | (1.4-1.9) | (3.2-4.8) | (0.21-0.49) | (0.00-0.27) | (0.23-0.72) |
|  |  |  | sd | 0.6 | 1.5 | 0.1 | 0.2 | 0.4 | 0.09 | 0.08 | 0.15 |
| 3 |  | 11 | mean | 7.5 | 78.6 | 7.6 | 1.7 | 4 | 0.34 | 0.05 | 0.38 |
|  | 35 |  | range | (6.7-8.5) | (76.3-80.9) | (7.4-7.7) | (1.4-1.9) | (3.1-4.8) | (0.18-0.45) | (0.00-0.20) | (0.18-0.58) |
|  |  |  | sd | 0.6 | 1.5 | 0.1 | 0.2 | 0.4 | 0.08 | 0.06 | 0.12 |
| 4 |  | 11 | mean | 7.5 | 78.8 | 7.6 | 1.7 | 4 | 0.34 | 0.05 | 0.39 |
|  | 35 |  | range | (6.8-8.2) | (76.2-81.1) | (7.4-7.7) | (1.4-1.9) | (3.3-4.8) | (0.21-0.47) | (0.00-0.31) | (0.22-0.73) |
|  |  |  | sd | 0.5 | 1.6 | 0.1 | 0.2 | 0.3 | 0.08 | 0.09 | 0.15 |
| 5 |  | 6 | mean | 6.5 | 75.1 | 7.7 | 1.9 | 4.2 | 0.31 | 0 | 0.31 |
|  | 42 |  | range | (6.3-6.7) | (73.3-76.2) | (7.6-8.0) | (1.8-1.9) | (3.9-4.8) | (0.26-0.43) | (0.00-0.02) | (0.26-0.43) |
|  |  |  | sd | 0.2 | 1 | 0.2 | 0.1 | 0.3 | 0.06 | 0.01 | 0.06 |

**Table S1.** (continued)

| 6 |  | 6 | mean | 6.4 | 75.3 | 7.7 | 1.8 | 4.2 | 0.31 | 0 | 0.31 |
| --- | --- | --- | --- | --- | --- | --- | --- | --- | --- | --- | --- |
|  | 42 |  | range | (6.2-6.6) | (74.5-76.0) | (7.6-7.7) | (1.8-1.9) | (3.8-4.8) | (0.26-0.42) | (0.00-0.02) | (0.26-0.42) |
|  |  |  | sd | 0.1 | 0.7 | 0 | 0.1 | 0.3 | 0.06 | 0.01 | 0.06 |
| 7 |  | 6 | mean | 6.4 | 74.9 | 7.7 | 1.9 | 4.2 | 0.31 | nn | 0.31 |
|  | 42 |  | range | (6.3-6.5) | (73.9-76.1) | (7.6-7.8) | (1.8-1.9) | (3.9-4.8) | (0.26-0.43) | --- | (0.26-0.43) |
|  |  |  | sd | 0.1 | 0.8 | 0.1 | 0.1 | 0.3 | 0.06 | --- | 0.06 |
| 8 |  | 6 | mean | 6.5 | 74.7 | 7.7 | 1.8 | 4.1 | 0.31 | 0.01 | 0.32 |
|  | 42 |  | range | (6.4-6.8) | (73.9-76.4) | (7.6-7.8) | (1.8-1.9) | (3.8-4.8) | (0.24-0.44) | (0.00-0.02) | (0.24-0.45) |
|  |  |  | sd | 0.2 | 0.9 | 0.1 | 0.1 | 0.3 | 0.07 | 0.01 | 0.08 |
| 9 |  | 20 | mean | 5.5 | 75.8 | 7.7 | 1.7 | 3.6 | 0.22 | 0.04 | 0.26 |
|  | 42 |  | range | (4.4-6.2) | (70.0-79.0) | (7.4-8.1) | (1.3-1.9) | (3.1-3.9) | (0.05-0.81) | (0.00-0.13) | (0.05-0.93) |
|  |  |  | sd | 0.4 | 2.2 | 0.2 | 0.2 | 0.3 | 0.18 | 0.04 | 0.21 |
| 10 |  | 18 | mean | 5.6 | 75.6 | 7.7 | 1.8 | 3.7 | 0.19 | 0.04 | 0.23 |
|  | 42 |  | range | (5.2-5.9) | (73.2-78.0) | (7.4-8.1) | (1.6-1.9) | (3.5-3.9) | (0.04-0.82) | (0.00-0.13) | (0.04-0.94) |
|  |  |  | sd | 0.2 | 1.6 | 0.2 | 0.1 | 0.1 | 0.19 | 0.04 | 0.22 |
| 11 |  | 10 | mean | 6.2 | 76.2 | 7.8 | 1.9 | 4 | 0.31 | 0.06 | 0.35 |
|  | 42 |  | range | (5.9-6.4) | (72.7-77.5) | (7.7-8.0) | (1.8-2.0) | (3.9-4.1) | (0.12-0.48) | (0.00-0.18) | (0.14-0.52) |
|  |  |  | sd | 0.1 | 1.4 | 0.1 | 0.1 | 0.1 | 0.78 | 0.05 | 0.86 |
| 12 |  | 11 | mean | 5.7 | 75.4 | 7.9 | 1.8 | 3.9 | 0.24 | 0.04 | 0.35 |
|  | 42 |  | range | (5.0-6.0) | (72.2-76.9) | (7.7-8.0) | (1.8-1.9) | (3.7-4.1) | (0.00-0.49) | (0.00-0.17) | (0.13-0.67) |
|  |  |  | sd | 0.3 | 1.4 | 0.1 | 0.1 | 0.1 | 0.17 | 0.05 | 0.17 |
|  |  |  | **mean** | **6.6** | **76.6** | **7.7** | **1.8** | **4.0** | **0.30** | **0.04** | **0.34** |
| **average** |  | **-** | **range** | **(4.4-8.6)** | **(70.0-81.8)** | **(7.3-8.1)** | **(1.3-2.0)** | **(3.1-4.8)** | **(0.00-0.82)** | **(0.00-0.33)** | **(0.04-0.94)** |
|  |  |  | **sd** | **1.3** | **4.8** | **0.5** | **0.5** | **1.0** | **0.87** | **0.19** | **0.98** |

T: operating temperature; n: number of weeks (monitored period), one analysis per reactor and week; mean: mean value; sd: standard deviation; TS: total solids; FM: fresh matter; DM: dry matter; VS: volatile solids; NH_4_-N: ammonium-bound nitrogen; N_tot_: total nitrogen, AA: acetic acid; PA: propionic acid; VFA: volatile fatty acids (sum of acetic acid, propionic acid and butyric acid comprising butyric, iso-butyric, caproic, valeric, and iso-valeric acid; total acids concentration is expressed as acetic acid equivalent)


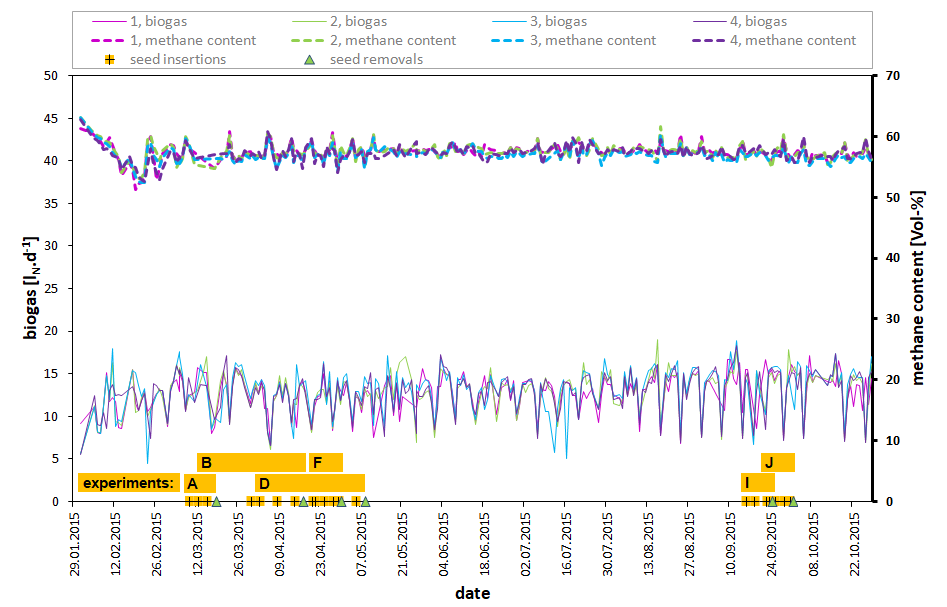


**Figure S1a**. Biogas yield (solid lines) and methane content (dashed, thick lines) during the operation of the lab-scale reactors 1-4 at 35°C. Above the x-axis, it is indicated when seeds were inserted or removed from the reactors (labeled as experiments A, B, D, F, I, and J).


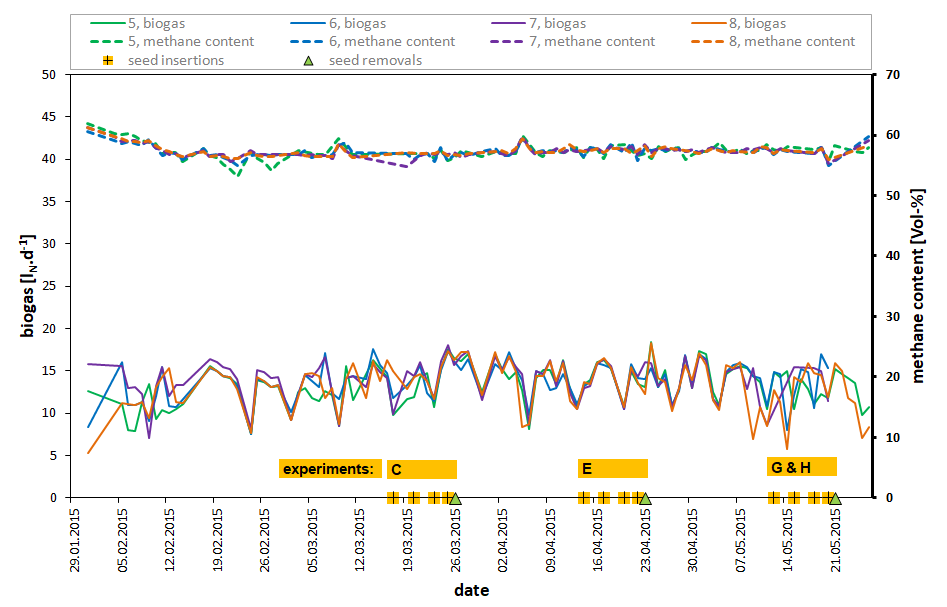


**Figure S1b**. Biogas yield (solid lines) and methane content (dashed, thick lines) during the operation of the lab-scale reactors 5-8 at 42°C. Above the x-axis, it is indicated when seeds were inserted or removed from the reactors (labeled as experiments C, E, G, and H).


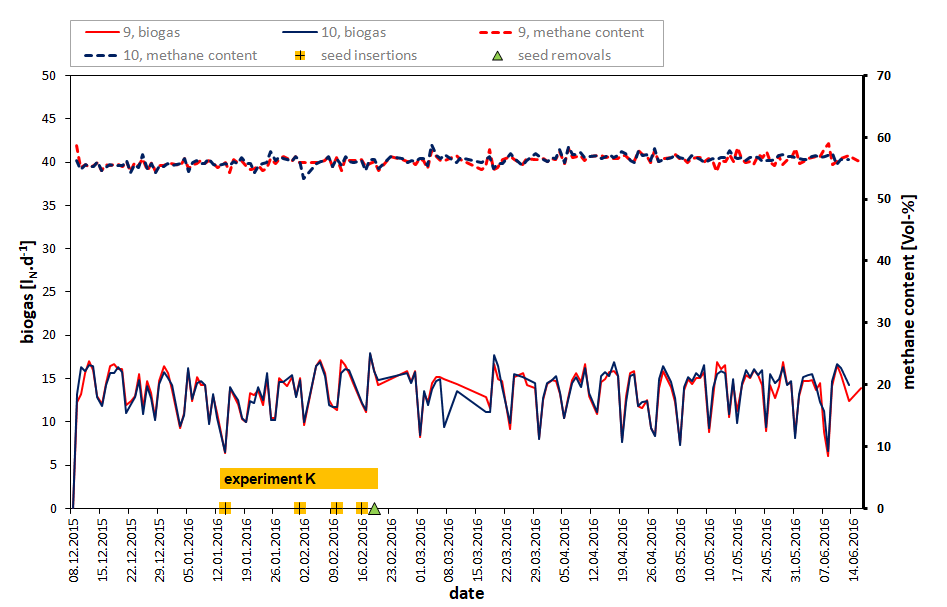


**Figure S1c**. Biogas yield (solid lines) and methane content (dashed, thick lines) during the operation of the lab-scale reactors 9 and 10 at 42°C. Above the x-axis, it is indicated when seeds were inserted or removed from the reactors (labeled as experiment K).


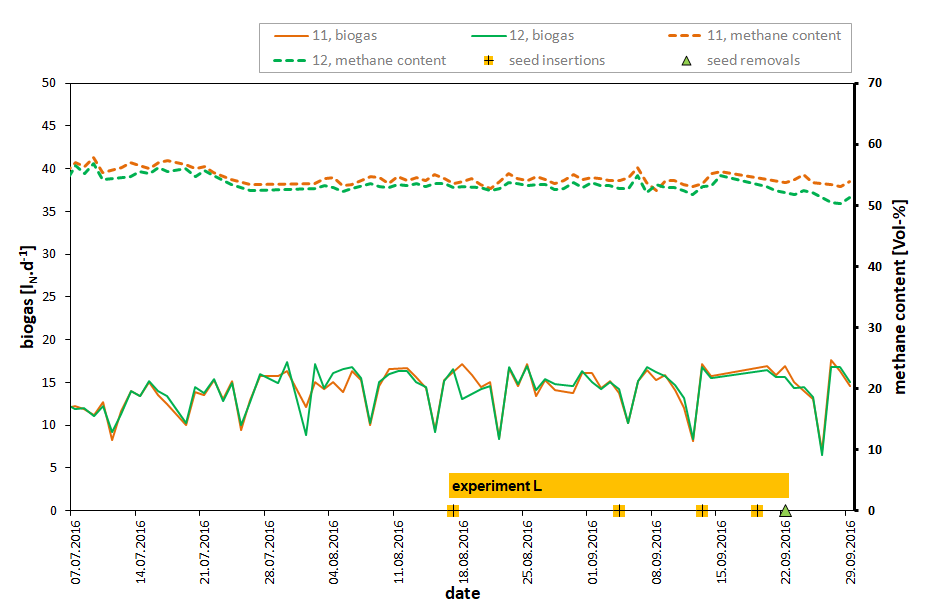


**Figure S1d**. Biogas yield (solid lines) and methane content (dashed, thick lines) during the operation of the lab-scale reactors 11 and 12 at 42°C. Above the x-axis, it is indicated when seeds were inserted or removed from the reactors (labeled as experiment L).
