# Supplementary figures and images for: Viability of Wildflower Seeds After Mesophilic Anaerobic Digestion in Lab-Scale Biogas Reactors
Source: Front Plant Sci. 2022 Jul 14;13:942346. doi: 10.3389/fpls.2022.942346 (PMC9337220; doi:10.3389/fpls.2022.942346)

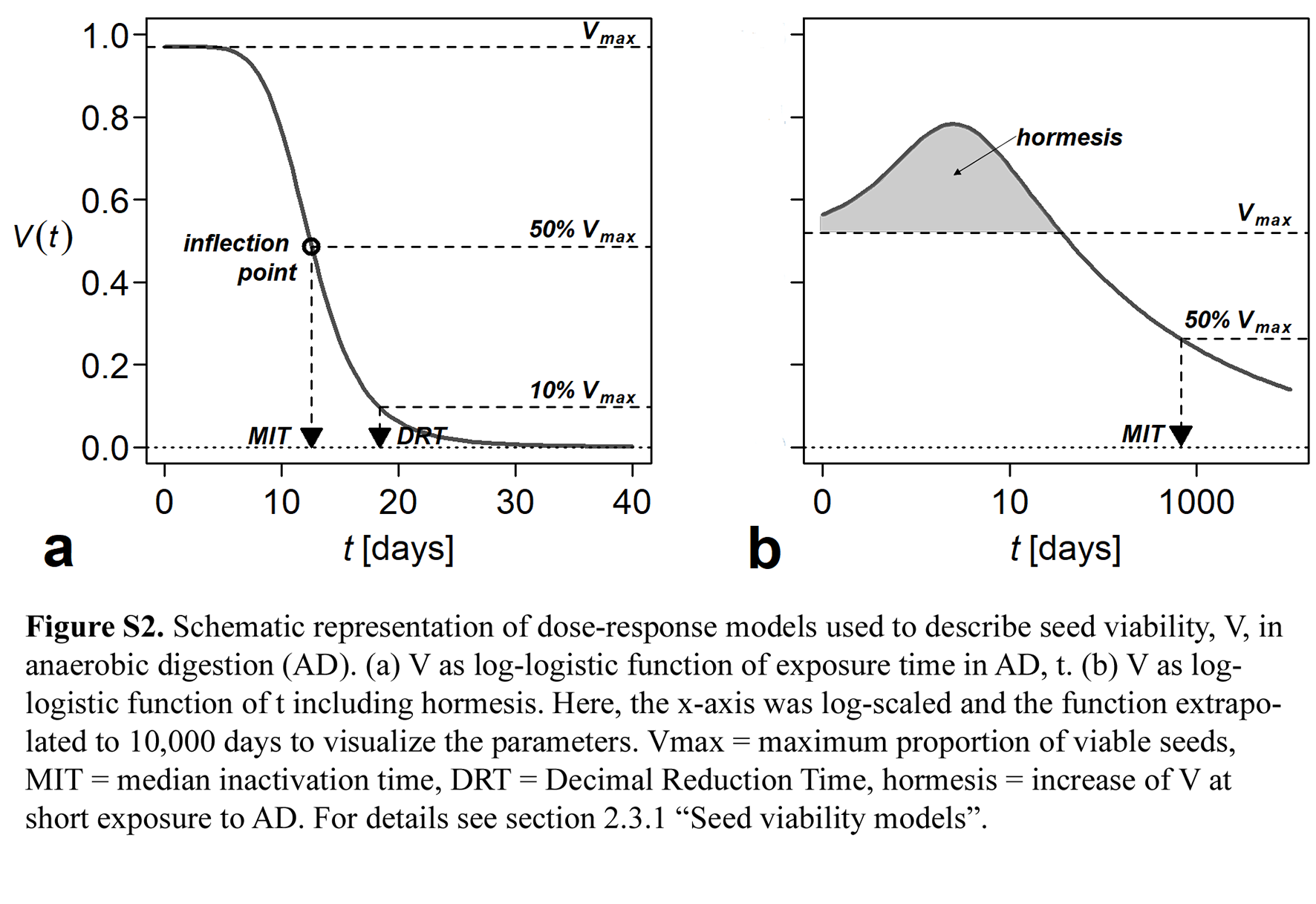

Supplement: Supplementary file 2 [file Image_2.tif]

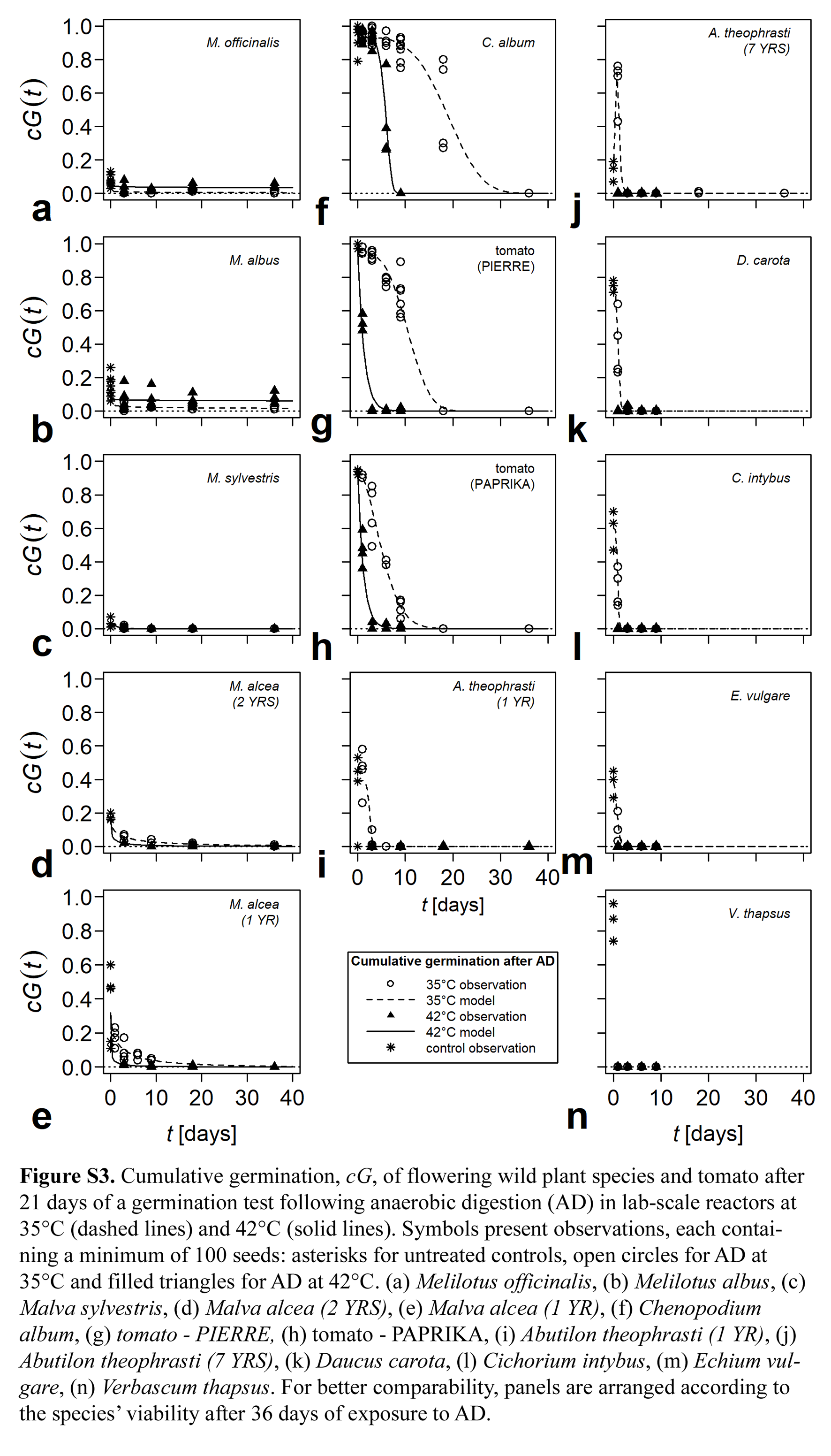

Supplement: Supplementary file 3 [file Image_3.tif]
