# Supplementary material for: Viability of Wildflower Seeds After Mesophilic Anaerobic Digestion in Lab-Scale Biogas Reactors
Source: Front Plant Sci. 2022 Jul 14;13:942346. doi: 10.3389/fpls.2022.942346 (PMC9337220; doi:10.3389/fpls.2022.942346)
Supplement: Supplementary file 4 [file Table_2.DOCX]

**Table S2.** Number of replicates and number of seeds per replicate for hardseeded (HS) and not hardseeded (NHS) species exposed mesophilic, anaerobic digestion (AD) at 35°C or 42°C for different durations (1 day – 36 days). The numbers of seeds per replicate are indicated by: # = 100 seeds, ## = 200 seeds, ### = 300 seeds.

|  | **Untreated control** |  | **AD 35°C** | | | | | |  | **AD 42°C** | | | | | |
| --- | --- | --- | --- | --- | --- | --- | --- | --- | --- | --- | --- | --- | --- | --- | --- |
|  |  |  | **1 d** | **3 d** | **6 d** | **9 d** | **18 d** | **36 d** |  | **1 d** | **3 d** | **6 d** | **9 d** | **18 d** | **36 d** |
| **HS species** |  |  |  |  |  |  |  |  |  |  |  |  |  |  |  |
| *Abutilon theophrasti – 7 YRS* | 3 # |  | 4 # | 8 # | 4 ## | 8 ## | 4 ## | 4 ### |  | 8 # | 8 # | 8 ## | 8 ### | - | - |
| *Abutilon theophrasti – 1 YR* | 4 # |  | 4 # | 4 # | 4 ## | 4 ### | - | - |  | - | 4 # | - | 4 # | 4 ## | 4 ### |
| *Malva alcea – 2 YRS* | 3 # |  | - | 4 # | - | 4 # | 4 ## | 4 ### |  | - | 2 # | - | 2 # | 2 ## | 2 ### |
| *Malva alcea – 1 YR* | 6 # |  | 4 # | 4 # | 4 ## | 4 ### | - | - |  | - | 4 # | - | 4 # | 4 ## | 4 ### |
| *Malva sylvestris* | 6 # |  | - | 4 # | - | 4 # | 4 ## | 4 ### |  | - | 4 # | - | 4 # | 4 ## | 4 ### |
| *Melilotus albus* | 9 # |  | - | 4 # | - | 4 # | 4 ## | 4 ### |  | - | 6 # | - | 6 # | 6 ## | 6 ### |
| *Melilotus officinalis* | 9 # |  | - | 4 # | - | 4 # | 4 ## | 4 ### |  | - | 4 # | - | 4 # | 4 ## | 4 ### |
|  |  |  |  |  |  |  |  |  |  |  |  |  |  |  |  |
| **NHS species** |  |  |  |  |  |  |  |  |  |  |  |  |  |  |  |
| *Chenopodium album* | 6 # |  | 4 # | 8 # | 4 ## | 8 ## | 4 ## | 4 ### |  | 4 # | 4 # | 4 ## | 4 ### | - | - |
| *Cichorium intybus* | 3 # |  | 4 # | 4 # | 4 ## | 4 ### | - | - |  | 4 # | 4 # | 4 ## | 4 ### | - | - |
| *Daucus carota* | 3 # |  | 4 # | 4 # | 4 ## | 4 ### | - | - |  | 4 # | 4 # | 4 ## | 4 ### | - | - |
| *Echium vulgare* | 3 # |  | 4 # | 4 # | 4 ## | 4 ### | - | - |  | 4 # | 4 # | 4 ## | 4 ### | - | - |
| *Verbascum thapsus* | 3 # |  | 4 # | 4 # | 4 ## | 4 ### | - | - |  | 4 # | 4 # | 4 ## | 4 ### | - | - |
| tomato – PAPRIKA | 3 # |  | 2 # | 4 # | 2 ## | 4 ## | 2 ## | 2 ### |  | 4 # | 4 # | 4 ## | 4 ### | - | - |
| tomato – PIERRE | 3 # |  | 4 # | 6 # | 4 ## | 6 ## | 2 ## | 2 ### |  | 4 # | 4 # | 4 ## | 4 ### | - | - |
