# Supplementary material for: Viability of Wildflower Seeds After Mesophilic Anaerobic Digestion in Lab-Scale Biogas Reactors
Source: Front Plant Sci. 2022 Jul 14;13:942346. doi: 10.3389/fpls.2022.942346 (PMC9337220; doi:10.3389/fpls.2022.942346)
Supplement: Supplementary file 5 [file Table_3.DOCX]

**Table S3.** Model type and fit (Chi²-test) and parameter estimates (standard errors in parentheses) obtained from the log-logistic (LL) and log-logistic models modified to capture hormesis (HLL) used to describe seed viability, *V*, during exposure to anaerobic digestion (AD) at 35°C and 42°C. The lower asymptote was set to zero for all models. Asterisks (*) indicate significant differences in parameter estimates between AD at 35°C and 42°C (p<0.05).

|  | **model** | |  | ***V_max_*** | |  | ***SLP*** | | | | |  | ***MIT* or *E*** | | | | |  | ***H*** | | | | |
| --- | --- | --- | --- | --- | --- | --- | --- | --- | --- | --- | --- | --- | --- | --- | --- | --- | --- | --- | --- | --- | --- | --- | --- |
|  | **type** | **p-value** |  |  |  |  | **35°C** | |  | **42°C** | |  | **35°C** | |  | **42°C** | |  | **35°C** | |  | **42°C** | |
| **HS species** |  |  |  |  |  |  |  |  |  |  |  |  |  |  |  |  |  |  |  |  |  |  |  |
| *Abutilon theophrasti – 7 YRS* | LL | <0.0001 |  | **0.78** | *(0.02)* |  | **2.31** | *(0.10)* |  | **-** | *-* |  | **1.30** | *(0.06)* |  | **-** | *-* |  | ***nd*** |  |  | ***nd*** |  |
| *Abutilon theophrasti – 1 YR* | LL | <0.0001 |  | **0.96** | *(0.01)* |  | **0.92** | *(0.06)* |  | **-** | *-* |  | **4.74** | *(0.24)* |  | **-** | *-* |  | ***nd*** |  |  | ***nd*** |  |
| *Malva alcea – 2 YRS* | LL | 0.0015 |  | **0.42** | *(0.01)* |  | **7.77** | *(3.92)* |  | **0.37** | *(0.12)* |  | **35.38** | *(0.65)* |  | **1.67** | *(1.36)* |  | ***nd*** |  |  | ***nd*** |  |
| *Malva alcea – 1 YR* | LL | 0.8024 |  | **0.51** | *(0.02)* |  | **0.81** | *(0.12)* | ***** | **0.38** | *(0.07)* |  | **5.10** | *(0.68)* |  | **0.06** | *(0.06)* |  | ***nd*** |  |  | ***nd*** |  |
| *Malva sylvestris* | HLL | 0.9893 |  | **0.26** | *(0.02)* |  | **1.23** | *(0.07)* |  | **1.21** | *(0.21)* |  | **1.89** | *(1.51)* |  | **7.16** | *(10.23)* |  | ***0.27*** | *(0.23)* |  | ***0.06*** | *(0.09)* |
| *Melilotus albus* | HLL | <0.0001 |  | **0.84** | *(0.01)* |  | **1.10** | *(0.01)* |  | **1.10** | *(0.02)* |  | **0.75** | *-* |  | **5.41** | *(0.82)* |  | ***1.16*** | *-* |  | ***0.18*** | *(0.03)* |
| *Melilotus officinalis* | HLL | 0.1823 |  | **0.85** | *(0.01)* |  | **1.00** | *(0.02)* | ***** | **1.05** | *(0.02)* |  | **1.57** | *(1.23)* |  | **1.52** | *(4.84)* |  | ***0.49*** | *(0.39)* |  | ***0.60*** | *(1.99)* |
|  |  |  |  |  |  |  |  |  |  |  |  |  |  |  |  |  |  |  |  |  |  |  |  |
| **NHS species** |  |  |  |  |  |  |  |  |  |  |  |  |  |  |  |  |  |  |  |  |  |  |  |
| *Chenopodium album* | LL | 0.9965 |  | **0.92** | *(0.00)* |  | **6.41** | *(0.28)* | ***** | **5.94** | *(0.29)* |  | **22.09** | *(0.30)* | *** | **6.77** | *(0.07)* |  | ***nd*** |  |  | ***nd*** |  |
| *Cichorium intybus* | LL | 1.0000 |  | **0.60** | *(0.03)* |  | **11.27** | *(28.91)* |  | **-** | *-* |  | **1.00** | *(0.02)* |  | **-** | *-* |  | ***nd*** |  |  | ***nd*** |  |
| *Daucus carota* | LL | <0.0001 |  | **0.80** | *(0.02)* |  | **3.03** | *(0.16)* |  | **-** | *-* |  | **1.32** | *(0.06)* |  | **-** | *-* |  | ***nd*** |  |  | ***nd*** |  |
| *Echium vulgare* | LL | 0.9999 |  | **0.41** | *(0.03)* |  | **3.90** | *(0.62)* |  | **-** | *-* |  | **0.90** | *(0.06)* |  | **-** | *-* |  | ***nd*** |  |  | ***nd*** |  |
| *Verbascum thapsus* ^a^ | - | - |  | **-** | *-* |  | **-** | *-* | **-** | **-** | *-* |  | **-** | *-* |  | **-** | *-* |  | ***nd*** |  |  | ***nd*** |  |
| tomato – PAPRIKA | LL | <0.0001 |  | **0.93** | *(0.01)* |  | **3.07** | *(0.18)* | ***** | **1.70** | *(0.08)* |  | **5.64** | *(0.18)* | *** | **1.26** | *(0.07)* |  | ***nd*** |  |  | ***nd*** |  |
| tomato – PIERRE | LL | 0.0136 |  | **0.97** | *(0.00)* |  | **5.79** | *(0.27)* | ***** | **1.63** | *(0.07)* |  | **12.56** | *(0.20)* | *** | **1.89** | *(0.09)* |  | ***nd*** |  |  | ***nd*** |  |

^a^ No model was fitted for *V. thapsus* because all seeds were inactivated before sampling at the first exposure time.

*V_max_*: maximum proportion of *V*; defined to be identical for both temperatures.

*SLP*: a parameter proportional to the slope of the curve in the inflection point.

MIT: mean inactivation time; i.e., the time after which the LL curve changes its flection and *V* is reduced to 50% of the initial *V*. In HLL, *E* is not directly interpretable.

*H*: hormesis effect size, which is not determined in LL models (“nd”).

-: parameters could not be estimated, mostly due to inactivation of seeds during the shortest exposure time.
