# Supplementary material for: Viability of Wildflower Seeds After Mesophilic Anaerobic Digestion in Lab-Scale Biogas Reactors
Source: Front Plant Sci. 2022 Jul 14;13:942346. doi: 10.3389/fpls.2022.942346 (PMC9337220; doi:10.3389/fpls.2022.942346)
Supplement: Supplementary file 6 [file Table_4.DOCX]

**Table S4.** Model type and fit (Chi²- test) and parameter estimates (standard errors in parentheses) of the Weibull (W) and log-logistic models modified to capture hormesis (HLL) used to describe cumulative germination, *cG*, during exposure to anaerobic digestion (AD) at 35°C and 42°C. The lower asymptote was set to zero for all models. Asterisks (*) indicate significant differences in parameter estimates between AD at 35°C and 42°C (p<0.05).

|  | **model** | |  | ***cG_max_*** | |  | ***SLP*** | | | | |  | ***IFT* or *E*** | | | | |  | ***H*** | | | | | | | |
| --- | --- | --- | --- | --- | --- | --- | --- | --- | --- | --- | --- | --- | --- | --- | --- | --- | --- | --- | --- | --- | --- | --- | --- | --- | --- | --- |
|  | **type** | **p-value** |  |  |  |  | **35°C** | |  | **42°C** | |  | **35°C** | |  | **42°C** | |  | **35°C** | | |  | | **42°C** | | |
| **HS species** |  |  |  |  |  |  |  |  |  |  |  |  |  |  |  |  |  |  |  |  | |  | |  | |  |
| *Abutilon theophrasti – 7 YRS* | HLL | <0.0001 |  | **0.14** | *(0.02)* |  | **6.09** | *(0.51)* |  | **-** | *-* |  | **1.10** | *(0.10)* |  | **-** | *-* |  | ***0.85*** | | *(0.21)* | |  | | ***-*** | *-* |
| *Abutilon theophrasti – 1 YR* | W | 0.7318 |  | **0.39** | *(0.02)* |  | **7.11** | *(4.71)* |  | **-** | *-* |  | **2.61** | *(0.24)* |  | **-** | *-* |  | ***nd*** | |  | |  | | ***nd*** |  |
| *Malva alcea – 2 YRS* | W | 0.9166 |  | **0.18** | *(0.02)* |  | **0.46** | *(0.09)* |  | **0.38** | *(0.14)* |  | **2.37** | *(1.08)* |  | **0.34** | *(0.45)* |  | ***nd*** | |  | |  | | ***nd*** |  |
| *Malva alcea – 1 YR* | W | 0.9602 |  | **0.32** | *(0.02)* |  | **0.53** | *(0.08)* |  | **0.30** | *(0.08)* |  | **2.27** | *(0.45)* |  | **0.07** | *(0.08)* |  | ***nd*** | |  | |  | | ***nd*** |  |
| *Malva sylvestris* | W | 1.0000 |  | **0.03** | *(0.01)* |  | **2.21** | *(10.88)* |  | **1.46** | *(10.60)* |  | **2.88** | *(0.89)* |  | **1.61** | *(7.30)* |  | ***nd*** | |  | |  | | ***nd*** |  |
| *Melilotus albus* | W | 0.7972 |  | **0.15** | *(0.01)* |  | **0.10** | *(0.02)* |  | **0.04** | *(0.03)* |  | **0.02** | *(0.03)* | *** | **1059.30** | *(2713.96)* |  | ***nd*** | |  | |  | | ***nd*** |  |
| *Melilotus officinalis* | W | 0.0272 |  | **0.07** | *(0.01)* |  | **0.12** | *(0.02)* |  | **0.10** | *(0.07)* |  | **0.01** | *(0.00)* | *** | **740.02** | *(1091.97)* |  | ***nd*** | |  | |  | | ***nd*** |  |
|  |  |  |  |  |  |  |  |  |  |  |  |  |  |  |  |  |  |  |  | |  | |  | |  |  |
| **NHS species** |  |  |  |  |  |  |  |  |  |  |  |  |  |  |  |  |  |  |  | |  | |  | |  |  |
| *Chenopodium album* | W | 0.9995 |  | **0.93** | *(0.00)* |  | **3.76** | *(0.24)* | ***** | **6.34** | *(1.22)* |  | **20.86** | *(0.35)* | *** | **6.23** | *(0.07)* |  | ***nd*** | |  | |  | | ***nd*** |  |
| *Cichorium intybus* | W | 1.0000 |  | **0.60** | *(0.03)* |  | **5.00** | *(302.21)* |  | **-** | *-* |  | **1.02** | *(1.17)* |  | **-** | *-* |  | ***nd*** | |  | |  | | ***nd*** |  |
| *Daucus carota* | W | 1.0000 |  | **0.75** | *(0.03)* |  | **3.10** | *(46.16)* |  | **-** | *-* |  | **1.15** | *(2.43)* |  | **-** | *-* |  | ***nd*** | |  | |  | | ***nd*** |  |
| *Echium vulgare* | W | 1.0000 |  | **0.38** | *(0.03)* |  | **2.24** | *(2.60)* |  | **-** | *-* |  | **0.99** | *(0.06)* |  | **-** | *-* |  | ***nd*** | |  | |  | | ***nd*** |  |
| *Verbascum thapsus*^a^ | - | - |  | **-** | *-* |  | **-** | *-* | **-** | **-** | *-* |  | **-** | *-* |  | **-** | *-* |  | ***-*** | |  | |  | | ***-*** |  |
| tomato – PAPRIKA | W | <0.0001 |  | **0.94** | *(0.01)* |  | **1.79** | *(0.11)* | ***** | **0.97** | *(0.05)* |  | **6.19** | *(0.16)* | *** | **1.19** | *(0.07)* |  | ***nd*** | |  | |  | | ***nd*** |  |
| tomato – PIERRE | W | <0.0001 |  | **0.95** | *(0.01)* |  | **3.26** | *(0.19)* | ***** | **1.03** | *(0.05)* |  | **11.81** | *(0.19)* | *** | **1.33** | *(0.07)* |  | ***nd*** | |  | |  | | ***nd*** |  |

^a^ No model was fitted for *V. thapsus* because all seeds failed to germinate before sampling at the first exposure time.

c*G_max_*: maximum proportion of *cG;* defined to be identical for both temperatures.

*SLP*: a parameter proportional to the slope of the curve in the inflection point.

*IFT*: inflection time; i.e., the time after which the W curve changes its flection. In HLL, *E* is not directly interpretable.

*H*: hormesis effect size, which is not determined in W models (“nd”).

-: parameters could not be estimated (-), mostly due to failure of germination during the shortest exposure time.
