# Supplementary material for: Viability of Wildflower Seeds After Mesophilic Anaerobic Digestion in Lab-Scale Biogas Reactors
Source: Front Plant Sci. 2022 Jul 14;13:942346. doi: 10.3389/fpls.2022.942346 (PMC9337220; doi:10.3389/fpls.2022.942346)
Supplement: Supplementary file 7 [file Table_5.DOCX]

**Table S5.** Estimated Decimal Reduction Times for cumulative Germination, *DRT*(*cG),* of flowering wild plant species and tomato after anaerobic digestion (AD) at 35°C or 42°C and difference between *DRTs* of *cG* and overall viability, *V* (see **Table 1**). Species are grouped according to their potential to exhibit hardseededness (HS) in their seeds or not (NHS). Standard errors of the mean are given in parentheses.

|  | ***DRT(cG)* [days]** | | | |  | ***DRT(cG*)-*DRT*(*V*) [days]** | |
| --- | --- | --- | --- | --- | --- | --- | --- |
|  | **AD 35°C** | | **AD 42°C** | |  | **AD 35°C** | **AD 35°C** |
| **HS species** |  |  |  |  |  |  |  |
| *Abutilon theophrasti – 7 YRS* | **2.6** | *(0.2)* | **<1** |  |  | -0.8 | nd |
| *Abutilon theophrasti – 1 YR* | **2.9** | *(0.1)* | **<1** |  |  | -48.9 | nd |
| *Malva alcea – 2 YRS* | **14.7** | *(2.9)* | **3.2** | *(1.8)* |  | -32.2 | -(365)^.^n |
| *Malva alcea – 1 YR* | **11.0** | *(1.5)* | **1.0** | *(0.6)* |  | -65.7 | -17.3 |
| *Malva sylvestris* | **4.2** | *(7.0)* | **2.9** | *(1.3)* |  | -(365)^.^n | -(365)^.^n |
| *Melilotus albus* | **61.6** | *(56.4)* | **>365** |  |  | -(365)^.^n | -(365)^.^n |
| *Melilotus officinalis* | **7.1** | *(5.9)* | **>365** |  |  | -(365)^.^n | -(365)^.^n |
|  |  |  |  |  |  |  |  |
| **NHS species** |  |  |  |  |  |  |  |
| *Chenopodium album* | **26.0** | *(0.7)* | **7.1** | *(0.2)* |  | -5.1 | -2.7 |
| *Cichorium intybus* | **1.2** | *(13.5)* | **<1** |  |  | 0 | nd |
| *Daucus carota* | **1.5** | *(9.2)* | **<1** |  |  | -1.2 | nd |
| *Echium vulgare* | **1.4** | *(0.6)* | **<1** |  |  | -0.2 | nd |
| *Verbascum thapsus* | **<1** |  | **<1** |  |  | nd | nd |
| tomato – PAPRIKA | **9.9** | *(0.3)* | **2.8** | *(0.1)* |  | -1.6 | -1.8 |
| tomato – PIERRE | **15.2** | *(0.4)* | **3.0** | *(0.1)* |  | -3.2 | -4.3 |

<1: seeds failed to germinate even after the shortest exposure time; no models fitted, no standard errors calculated.

>365: estimated *DRT* values exceeded one year (365 days); no models fitted, no standard errors calculated.

nd: The difference between *DRT(cG)* and *DRT(V)* was not determined if no models could be fitted for *cG*, *V* or both.

-(365)^.^n: Differences between *DRT(cG)* and *DRT(V)* exceeding one year.
